# Synthesis, antimicrobial, anti-biofilm evaluation, and molecular modelling study of new chalcone linked amines derivatives

**DOI:** 10.1080/14756366.2018.1461855

**Published:** 2018-05-03

**Authors:** Shahenda M. El-Messery, El-Sayed E. Habib, Sarah T. A. Al-Rashood, Ghada S. Hassan

**Affiliations:** a Department of Pharmaceutical Organic Chemistry, Faculty of Pharmacy, Mansoura University, Mansoura, Egypt;; b Department of Pharmaceutics and Pharmaceutical Technology, College of Pharmacy, Taibah University, Al-Madinah Al-Munawarah, Kingdom of Saudi Arabia;; c Department of Microbiology, Faculty of Pharmacy, Mansoura University, Mansoura, Egypt;; d Department of Pharmaceutical Chemistry, College of Pharmacy, King Saud University, Riyadh, Saudi Arabia;; e Department of Medicinal Chemistry, Faculty of Pharmacy, Mansoura University, Mansoura, Egypt

**Keywords:** Chalcones linked amines, antimicrobial/anti-biofilm activity, c-di-GMP inhibition, molecular modelling

## Abstract

A series of amide chalcones conjugated with different secondary amines were synthesised and characterised by different spectroscopic techniques ^1^H NMR, ^13^C NMR, and ESI-MS. They were screened for *in vitro* antibacterial activity. Compounds **36**, **37**, **38**, **42**, and **44** are the most active among the synthesised series exhibiting MIC value of 2.0–10.0 µg/ml against different bacterial strains. Compound **36** was equipotent to the standard drug Ampicillin displaying MBC value of 2.0 µg/ml against the bacterial strain *Staphylococcus aureus.* The products were screened for anti-biofilm activity. Compounds **36**, **37**, and **38** exhibited promising anti-biofilm activity with IC_50_ value ranges from 2.4 to 8.6 µg. Molecular modelling was performed suggesting parameters of signalling anti-biofilm mechanism. AspB327 HisB340 (arene–arene interaction) and IleB328 amino acid residues seemed of higher importance to inhibit c-di-GMP. Hydrophobicity may be crucial for activity. ADME calculations suggested that compounds **36**, **37**, and **38** could be used as good orally absorbed anti-biofilm agents.

## Graphical abstract

**Figure:**
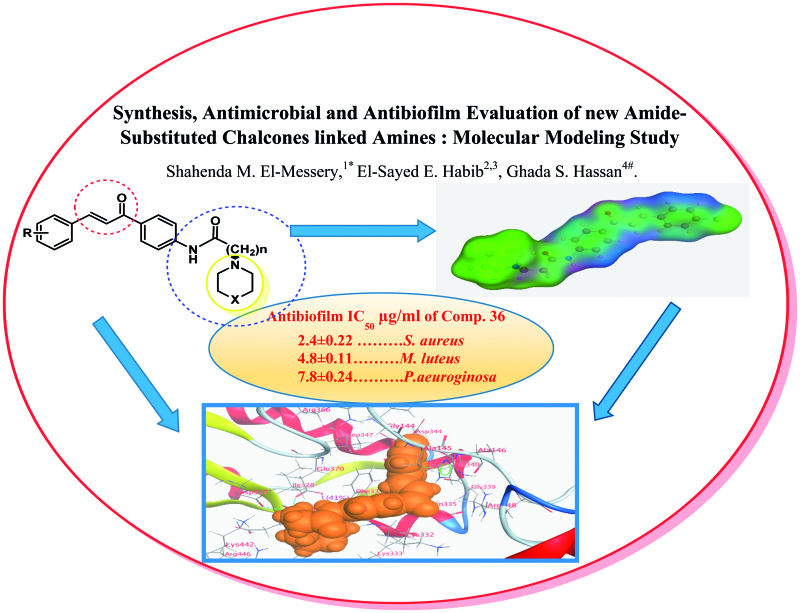

## Introduction

Human struggle against the affliction of infectious diseases is eternal. The contemporary treatment of infectious diseases involves administration of a multidrug regimen over a long period of time has led to the rapid emergence of multidrug-resistant strains plus a high level of patient non-compliance^1^ ^,^ ^2^. Biofilms are multicellular bacterial communities encased in an extracellular matrix. Biofilms have been estimated by the National Institutes of Health to be associated with 80% of all bacterial infections^3^ ^,^ ^4^. It was recently estimated that biofilm-based disease is responsible for 19 million infections annually in the US, resulting in hundreds of thousands of fatalities and billions of dollars in medical expenses^5^. Increased antibiotic tolerance has been promoted by biofilm formation to levels 1000 times greater than those observed in planktonic bacteria. Moreover, chronic infections, such as lung pneumonia of cystic fibrosis patients, otitis media, non-healing wounds, and contamination of artificial medical implants, are also associated with biofilm formation that leads to inefficient treatment of these infections.

The second messenger cyclic di-GMP (c-di-GMP) has recently emerged as a novel signal that controls biofilm formation and represses motility. Synthesis of c-di-GMP occurs via diguanylate cyclase (DGC) enzymes encoding GGDEF domains, while degradation of c-di-GMP occurs via phosphodiesterase (PDE) enzymes^6^ ^,^ ^7^. Analysis of bacterial genome sequences revealed that enzymes predicted to synthesize or degrade c-diGMP are found in 85% of all bacteria, including many prominent human pathogens. Deletion of active DGCs completely abolishes biofilm formation, suggesting c-di-GMP is essential for this process in bacteria that utilize the signal^8–11^.

**Chart 1.**
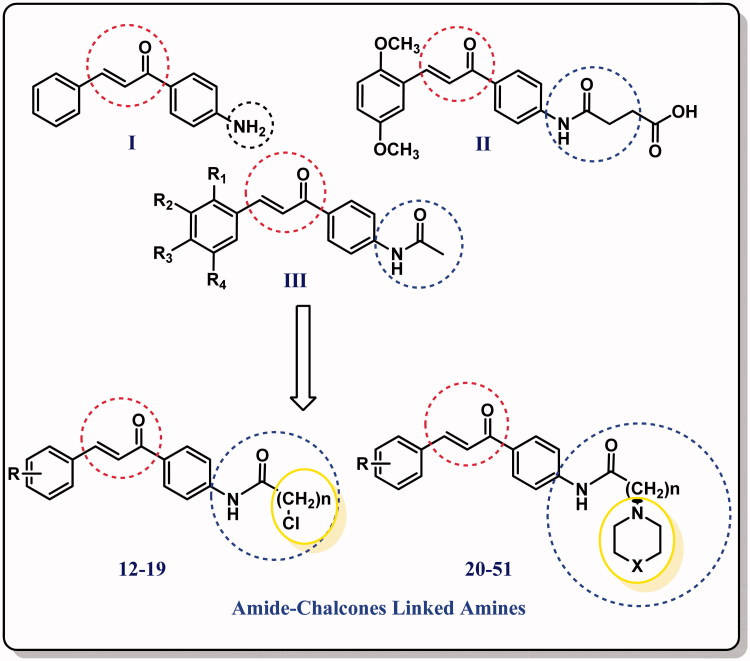
The rational design of our synthesised compounds.

Chalcone scaffold represents a core unit that exhibits various biological activities especially highlighting antimicrobial activity. In addition, they present a combinatorial assembly for the synthesis of heterocyclic scaffolds^12–14^. Molecular hybridisation is a new concept in drug design and development based on the combination of pharmacophoric moieties of different bioactive substances to produce a new hybrid compound with improved affinity and efficacy when compared to the parent drugs. Additionally, this strategy can result in compounds presenting modified selectivity profile, different and/or dual modes of action and reduced undesired side^15^ ^,^ ^16^. Based on literature survey focusing on both amines and chalcone in one hybrid structure, several derivatives have been identified as potent antimicrobials (Chart 1) and taken as lead compounds for further optimisation^17–20^.

Taking all together and as part of on-going research work aimed to the development of small molecules as therapeutic agents^21–24^, we have managed to design new amide chalcones/amine hybrids with expected high antimicrobial activity^25^ ^,^ ^26^. Despite the advances in antimicrobial activity evaluation, potent biofilm modulators are still sorely underdeveloped. So herein we report the discovery of a few novel derivatives that possess anti-biofilm activity. In addition, molecular modelling studies are performed to get into a molecular in-depth level. ci-diGMP was targeted as the major cause for biofilm formation to search for the suggested mechanism for the anti-biofilm obtained activity which could pave the way for further anti-biofilm drug discovery.

## Experimental section

The synthesis of the designed compounds was performed in Faculty of Pharmacy, Mansoura University, Mansoura, Egypt. The *in vitro* antimicrobial screening was conducted in the Department of Pharmaceutics and Pharmaceutical Technology (Microbiology), College of Pharmacy, Taibah University, Almadinah Almunawarah, Saudi Arabia. Molecular docking experiments were performed using “Molecular Operating Environment” software on Core i7 workstation. Melting points (°C) were determined on Mettler FP80 melting point apparatus and are uncorrected. All of the new compounds were analysed for C, H and N and agreed with the proposed structures within ±0.4% of the theoretical values. ^1^H- and ^13^C-NMR were recorded on a Bruker 500 MHz FT spectrometer (Bruker Daltonics Inc., Billerica, MA); chemical shifts are expressed in δ ppm with reference to TMS. Mass spectral (MS) data were obtained on a Perkin Elmer, Clarus 600 GC/MS and Joel JMS-AX 500 mass spectrometers (Perkin Elmer, Waltham, MA). Thin layer chromatography was performed on pre-coated (0.25 mm) silica gel GF_254_ plates (E. Merck, Germany), compounds were detected with 254 nm UV lamp. Silica gel (60–230 mesh) was employed for routine column chromatography separations. All the fine chemicals and reagents used were purchased from Aldrich Chemicals Co. (St. Louis, MO). Copies of ^1^H-NMR and ^13^C-NMR spectra of synthesized compounds are reported in the Supplementary data.

### Chemistry

#### (E)-2-chloro-N-(4-(3-(substituedphenyl)acryloyl)phenyl)acetamides (12–15), (E)-3-chloro-N-(4-(3-(substitutedphenyl)acryloyl)phenyl)propanamides (16–19)

A mixture of 1-(4-aminophenyl)-3-(substitutedphenyl)prop-2-en-1-ones **6–9** (0.01 mol), potassium carbonate (2.07 g, 0.015 mol) in dry toluene (50 ml) was stirred at room temperature, while chloroacetyl chloride (**10**, 1.7 g, 1.2 ml, 0.015 mol) or 3-chloropropionyl chloride (**11**, 1.9 g, 1.4 ml, 0.015 mol), was added dropwise. Stirring continued for 36 h, solvent was then removed *in vacuo* and the residue obtained was triturated with water, filtered, dried, and recrystallised (Table 1).

**Table 1.** Physicochemical properties of the newly synthesised compounds **12**–**19** and **20**–**51.**


Compound No.	R	X	*n*	Yield %	m.p. °C	Molecular formulae^a^
**12**	4-Cl	–	1	68	119–121	C_17_H_13_Cl_2_NO_2_
**13**	4-OCH_3_	–	1	72	137–139	C_18_H_16_ClNO_3_
**14**	3,4-di(OCH_3_)	–	1	61	141–143	C_19_H_18_ClNO_4_
**15**	3,4,5-tri(OCH_3_)	–	1	52	129–133	C_20_H_20_ClNO_5_
**16**	4-Cl	–	2	72	133–136	C_18_H_15_Cl_2_NO_2_
**17**	4-OCH_3_	–	2	49	107–109	C_19_H_18_ClNO_3_
**18**	3,4-di(OCH_3_)	–	2	38	133–136	C_20_H_20_ClNO_4_
**19**	3,4,5-tri(OCH_3_)	–	2	69	136–139	C_21_H_22_ClNO_5_
**20**	4-Cl	CH_2_	1	43	149–151	C_22_H_23_ClN_2_O_2_
**21**	4-OCH_3_	CH_2_	1	54	211–214	C_23_H_26_N_2_O_3_
**22**	3,4-di(OCH_3_)	CH_2_	1	40	157–160	C_24_H_28_N_2_O_4_
**23**	3,4,5-tri(OCH_3_)	CH_2_	1	39	163–168	C_25_H_30_N_2_O_5_
**24**	4-Cl	O	1	51	142–146	C_21_H_21_ClN_2_O_3_
**25**	4-OCH_3_	O	1	63	182–184	C_22_H_24_N_2_O_4_
**26**	3,4-di(OCH_3_)	O	1	45	151–154	C_23_H_26_N_2_O_5_
**27**	3,4,5-tri(OCH_3_)	O	1	70	154–159	C_24_H_28_N_2_O_6_
**28**	4-Cl	N-CH_3_	1	56	173–177	C_22_H_24_ClN_3_O_2_
**29**	4-OCH_3_	N-CH_3_	1	62	191–193	C_23_H_27_N_3_O_3_
**30**	3,4-di(OCH_3_)	N-CH_3_	1	80	184–188	C_24_H_29_N_3_O_4_
**31**	3,4,5-tri(OCH_3_)	N-CH_3_	1	59	161–164	C_25_H_31_N_3_O_5_
**32**	4-Cl	N-C_6_H_5_	1	73	168–170	C_27_H_26_ClN_3_O_2_
**33**	4-OCH_3_	N-C_6_H_5_	1	52	146–149	C_28_H_29_N_3_O_3_
**34**	3,4-di(OCH_3_)	N-C_6_H_5_	1	60	172–178	C_29_H_31_N_3_O_4_
**35**	3,4,5-tri(OCH_3_)	N-C_6_H_5_	1	48	169–172	C_30_H_33_N_3_O_5_
**36**	4-Cl	CH_2_	2	39	154–157	C_23_H_25_ClN_2_O_2_
**37**	4-OCH_3_	CH_2_	2	61	135–139	C_24_H_28_N_2_O_3_
**38**	3,4-di(OCH_3_)	CH_2_	2	89	166–169	C_25_H_30_N_2_O_4_
**39**	3,4,5-tri(OCH_3_)	CH_2_	2	58	188–193	C_26_H_32_N_2_O_5_
**40**	4-Cl	O	2	37	182–186	C_22_H_23_ClN_2_O_3_
**41**	4-OCH_3_	O	2	74	125–128	C_23_H_26_N_2_O_4_
**42**	3,4-di(OCH_3_)	O	2	84	226–229	C_24_H_28_N_2_O_5_
**43**	3,4,5-tri(OCH_3_)	O	2	52	195–199	C_25_H_30_N_2_O_6_
**44**	4-Cl	N-CH_3_	2	72	204–208	C_23_H_26_ClN_3_O_2_
**45**	4-OCH_3_	N-CH_3_	2	74	158–163	C_24_H_29_N_3_O_3_
**46**	3,4-di(OCH_3_)	N-CH_3_	2	63	194–198	C_25_H_31_N_3_O_4_
**47**	3,4,5-tri(OCH_3_)	N-CH_3_	2	69	234–238	C_26_H_33_N_3_O_5_
**48**	4-Cl	N-C_6_H_5_	2	54	149–153	C_28_H_28_ClN_3_O_2_
**49**	4-OCH_3_	N-C_6_H_5_	2	49	142–145	C_29_H_31_N_3_O_3_
**50**	3,4-di(OCH_3_)	N-C_6_H_5_	2	62	182–187	C_30_H_33_N_3_O_4_
**51**	3,4,5-tri(OCH_3_)	N-C_6_H_5_	2	58	226–232	C_31_H_35_N_3_O_5_

a Analysed for C,H,N; results were within ±0.4% of the theoretical values for the formulae given.

#### (E)-2-Chloro-N-(4-(3-(4-chlorophenyl)acryloyl)phenyl)acetamide (12)

^1^H-NMR (DMSO-d_6_) δ 3.62 (brs, 1H, NH), 4.56 (s, 2H, CH_2_), 7.53 (d, 2H, *J* = 8.5 Hz, Ar-H), 7.82 (d, 2H, *J* = 8.5 Hz, Ar-H), 7.93 (d, 2H, *J* = 8.5 Hz, Ar-H), 7.96 (s, 1H, Olefinic-H), 8.00 (s, 1H, Olefinic-H), 8.18 (d, 2H, *J* = 8.0 Hz, Ar-H). ^13^C-NMR δ 40.1, 43.5, 118.7, 122.7, 128.9, 129.9, 130.5, 131.5, 132.7, 133.7, 133.9, 134.9, 137.6, 141.9, 143.0, 165.3, 187.5. **MS** *m/z* (%): 333.2 (12.0, M^+^).

#### (E)-2-Chloro-N-(4-(3-(4-methoxyphenyl)acryloyl)phenyl)acetamide (13)

^1^H-NMR (DMSO-d_6_) δ 3.84 (s, 3H, OCH_3_), 4.28 (s, 2H, CH_2_), 7.04 (d, 2H, *J* = 9.0 Hz, Ar-H), 7.72 (s, 1H, Olefinic-H), 7.80 (s, 1H, Olefinic-H), 7.82 (d, 2H, *J* = 6.0 Hz, Ar-H), 7.85 (d, 2H, *J* = 9.0 Hz, Ar-H), 8.15 (d, 2H, *J* = 8.5 Hz, Ar-H), 10.85 (brs, 1H, NH). ^13^C-NMR δ 40.1, 55.4, 114.4, 118.7, 120.3, 121.8, 128.6, 129.7, 130.7, 131.0, 132.9, 133.7, 139.7, 141.0, 145.7, 146.2, 166.8, 189.3. **MS** *m/z* (%): 329.78 (27.4, M^+^).

#### (E)-2-Chloro-N-(4-(3-(3,4-dimethoxyphenyl)acryloyl)phenyl)acetamide (14)

^1^H-NMR (DMSO-d_6_) δ 3.83 (s, 3H, OCH_3_), 3.88 (s, 3H, OCH_3_), 4.39 (s, 2H, CH_2_), 7.03 (d, 2H, *J* = 8.0 Hz, Ar-H), 7.38 (dd, 2H, *J* = 1.0, 1.5 Hz, Ar-H), 7.54 (s, 1H, Ar-H), 7.68 (s, 1H, Olefinic-H), 7.71 (s, 1H, Olefinic-H), 8.17 (d, 2H, *J* = 7.5 Hz, Ar-H), 11.09 (s, 1H, NH). ^13^C-NMR δ 40.6, 40.9, 43.6, 55.6, 55.8, 110.7, 111.6, 118.7, 119.5, 123.9, 127.6, 129.8, 132.9, 134.7, 136.2, 143.9, 149.0, 151.2, 187.5. **MS** *m/z* (%): 359.8 (9.4, M^+^).

#### (E)-2-Chloro-N-(4-(3-(3,4,5-trimethoxyphenyl)acryloyl)phenyl)acetamide (15)

^1^H-NMR (DMSO-d_6_) δ 3.73 (s, 3H, OCH_3_), 3.88 (s, 3H, OCH_3_), 4.40 (s, 3H, OCH_3_), 4.56 (s, 2H, CH_2_), 7.24 (s, 1H, Ar-H), 7.68 (s, 1H, Olefinic-H), 7.71 (s, 1H, Olefinic-H), 7.85 (d, 2H, *J* = 8.5 Hz, Ar-H), 7.92 (s, 1H, Ar-H), 8.21 (d, 2H, *J* = 8.5 Hz, Ar-H), 11.09 (s, 1H, NH). ^13^C-NMR δ 41.5, 43.6, 55.7, 56.1, 60.1, 106.5, 107.2, 118.7, 121.1, 129.8, 130.3, 132.7, 139.7, 142.8, 143.0, 143.9, 153.1, 165.4, 167.1, 187.6. **MS** *m/z* (%): 389.8 (15.9, M^+^).

#### (E)-3-Chloro-N-(4-(3-(4-chlorophenyl)acryloyl)phenyl)propanamide (16)

^1^H-NMR (DMSO-d_6_) δ 2.92 (t, 2H, *J* = 12.5 Hz, CH_2_), 3.91 (t, 2H, *J* = 12.5 Hz, CH_2_), 7.55 (d, 2H, *J* = 8.5 Hz, Ar-H), 7.93 (s, 1H, Olefinic-H), 7.96 (s, 1H, Olefinic-H), 7.97 (d, 2H, *J* = 8.0 Hz, Ar-H), 8.00 (d, 2H, *J* = 8.5 Hz, Ar-H), 8.18 (d, 2H, *J* = 8.0 Hz, Ar-H), 10.70 (s, 1H, NH). ^13^C-NMR δ 40.1, 40.5, 118.8, 122.8, 127.6, 128.3, 128.9, 129.9, 130.5, 130.9, 131.7, 132.2, 133.8, 134.9, 141.9, 143.5, 163.7, 187.4. **MS** *m/z* (%): 347.2 (20.3, M^+^).

#### (E)-3-Chloro-N-(4-(3-(4-methoxyphenyl)acryloyl)phenyl)propanamide (17)

^1^H-NMR (DMSO-d_6_) δ 2.93 (t, 2H, *J* = 12.5 Hz, CH_2_), 3.83 (s, 3H, OCH_3_), 3.90 (t, 2H, *J* = 12.5 Hz, CH_2_), 7.02 (d, 2H, *J* = 9.0 Hz, Ar-H), 7.68 (s, 1H, Olefinic-H), 7.72 (s, 1H, Olefinic-H), 7.82 (d, 2H, *J* = 6.5 Hz, Ar-H), 7.85 (d, 2H, *J* = 8.5 Hz, Ar-H), 8.14 (d, 2H, *J* = 9.0 Hz, Ar-H), 10.63 (s, 1H, NH). ^13^C-NMR δ 40.1, 40.6, 55.4, 114.4, 118.5, 119.5, 120.3, 122.7, 124.1, 125.9, 127.4, 129.7, 130.7, 132.6, 143.2, 143.3, 161.3, 168.6, 187.4. **MS** *m/z* (%): 343.8 (6.1, M^+^).

#### (E)-3-Chloro-N-(4-(3-(3,4-dimethoxyphenyl)acryloyl)phenyl)propanamide (18)

^1^H-NMR (DMSO-d_6_) δ 2.92 (t, 2H, *J* = 12.5 Hz, CH_2_), 3.83 (s, 3H, OCH_3_), 3.88 (s, 3H, OCH_3_), 3.91 (t, 2H, *J* = 12.5 Hz, CH_2_), 7.04 (d, 2H, *J* = 8.5 Hz, Ar-H), 7.38 (dd, 2H, *J* = 1.5, 1.5 Hz, Ar-H), 7.55 (s, 1H, Ar-H), 7.68 (s, 1H, Olefinic-H), 7.71 (s, 1H, Olefinic-H), 7.84 (d, 2H, *J* = 8.0 Hz, Ar-H), 10.63 (s, 1H, NH). ^13^C-NMR δ 40.1, 40.6, 55.6, 55.8, 110.7, 111.6, 118.4, 118.7, 119.5, 123.8, 127.6, 129.7, 131.7, 132.6, 143.2, 143.8, 149.0, 151.2, 168.6, 187.4. **MS** *m/z* (%): 373.8 (14.9, M^+^).

#### (E)-3-Chloro-N-(4-(3-(3,4,5-trimethoxyphenyl)acryloyl)phenyl)propanamid (19)

^1^H-NMR (DMSO-d_6_) δ 2.93 (t, 2H, *J* = 12.5 Hz, CH_2_), 3.65 (s, 3H, OCH_3_), 3.73 (s, 3H, OCH_3_), 3.88 (s, 3H, OCH_3_), 3.91 (t, 2H, *J* = 12.5 Hz, CH_2_), 7.23 (s, 1H, Ar-H), 7.67 (s, 1H, Olefinic-H), 7.70 (s, 1H, Olefinic-H), 7.85 (d, 2H, *J* = 8.5 Hz, Ar-H), 7.89 (s, 1H, Ar-H), 8.18 (dd, 2H, *J* = 5.5, 5.5 Hz, Ar-H), 10.68 (s, 1H, NH). ^13^C-NMR δ 40.1, 40.6, 55.7, 56.1, 60.1, 106.5, 118.4, 118.7, 121.1, 127.6, 129.8, 130.3, 131.7, 132.4, 139.7, 143.4, 143.9, 153.1, 163.7, 168.7, 187.5. **MS** *m/z* (%): 403.8 (5.1, M^+^).

#### (E)-N-(4-(3-(substitutedphenyl)acryloyl)phenyl)-2-(substituted)acetamides (20–35), (E)-N-(4-(3-(substitutedphenyl)acryloyl)phenyl)-3-(substituted)propanamides (36–51)

To a stirred solution of **12–19** (0.01 mol) in dry toluene (50 ml), the appropriate amine (0.04 mol) was added dropwise. The reaction mixture was heated under reflux for 3–5 h. Solvent was then distilled under reduced pressure, the obtained residue was triturated with ice-water, filtered, dried, and recrystallised (Table 1).

#### (E)-N-(4-(3-(4-Chlorophenyl)acryloyl)phenyl)-2-(piperidin-1-yl)acetamide (20)

^1^H-NMR (CDCl_3_-d_6_) δ 1.58–2.18 (m, 6H, piperidine-H), 2.58 (t, 2H, piperidine-H), 2.84 (t, 2H, piperidine-H), 3.18 (s, 2H, CH_2_), 7.40 (d, 2H, *J* = 9.0 Hz, Ar-H), 7.49 (s, 1H, Olefinic-H), 7.52 (s, 1H, Olefinic-H), 7.59 (d, 2H, *J* = 7.0 Hz, Ar-H), 7.74 (d, 2H, *J* = 8.5 Hz, Ar-H), 7.82 (brs, 1H, NH), 8.02 (d, 2H, *J* = 7.0 Hz, Ar-H). ^13^C-NMR δ 21.3, 22.7, 25.8, 54.5, 56.3, 62.4, 119.2, 122.2, 128.5, 129.3, 129.6, 130.0, 133.5, 133.8, 135.1, 136.0, 136.4, 141.9, 142.9, 143.0, 168.2, 188.7. **MS** *m/z* (%): 382.8 (5.0, M^+^).

#### (E)-N-(4-(3-(4-Methoxyphenyl)acryloyl)phenyl)-2-(piperidin-1-yl)acetamide (21)

^1^H-NMR (CDCl_3_-d_6_) δ 1.61–1.94 (m, 6H, piperidine-H), 3.16 (s, 2H, CH_2_), 3.75 (t, 2H, *J* = 1.5 Hz, piperidine-H), 3.80 (t, 2H, piperidine-H), 3.86 (s, 3H, OCH_3_), 6.94 (d, 2H, *J* = 9.5 Hz, Ar-H), 7.40 (s, 1H, Olefinic-H), 7.43 (s, 1H, Olefinic-H), 7.60 (d, 2H, *J* = 7.5 Hz, Ar-H), 7.80 (d, 2H, *J* = 8.0 Hz, Ar-H), 7.86 (brs, 1H, NH), 8.02 (d, 2H, *J* = 7.0 Hz, Ar-H). ^13^C-NMR δ 24.1, 25.3, 25.7, 55.3, 55.5, 56.7, 113.7, 114.4, 116.0, 116.8, 118.2, 119.3, 119.5, 122.2, 127.7, 129.6, 129.8, 130.3, 131.6, 141.5, 144.5, 161.7, 189.1. **MS** *m/z* (%): 378.4 (31.6, M^+^).

#### (E)-N-(4-(3-(3,4-Dimethoxyphenyl)acryloyl)phenyl)-2-(piperidin-1-yl)acetamide (22)

^1^H-NMR (CDCl_3_-d_6_) δ 1.61–3.18 (m, 10H, piperidine-H), 3.21(s, 2H, CH_2_), 3.86 (s, 3H, OCH_3_), 3.94 (s, 3H, OCH_3_), 7.11 (d, 2H, *J* = 8.5 Hz, Ar-H), 7.45 (s, 1H, Olefinic-H), 7.51 (s, 1H, Olefinic-H), 7.63 (s, 1H, Ar-H), 7.88 (d, 2H, *J* = 8.0 Hz, Ar-H), 7.95 (d, 2H, *J* = 7.0 Hz, Ar-H), 10.98 (brs, 1H, NH). ^13^C-NMR δ 23.8, 24.1, 25.3, 25.9, 53.2, 54.6, 56.8, 63.1, 110.2, 111.4, 118.3, 119.3, 121.4, 122.9, 128.0, 128.9, 129.9, 135.6, 137.3, 139.5, 146.0, 156.3, 168.2, 188.4. **MS** *m/z* (%): 408.5 (22.6, M^+^).

#### (E)-2-(Piperidin-1-yl)-N-(4-(3-(3,4,5-trimethoxyphenyl)acryloyl)phenyl)acetamide (23)

^1^H-NMR (CDCl_3_-d_6_) δ 1.57–3.06 (m, 10H, piperidine-H), 3.71 (s, 2H, CH_2_), 3.88 (s, 3H, OCH_3_), 3.91 (s, 3H, OCH_3_), 4.00 (s, 3H, OCH_3_), 7.32 (s, 1H, Olefinic-H), 7.40 (s, 1H, Olefinic-H), 7.51 (s, 1H, Ar-H), 7.74 (d, 2H, *J* = 8.5 Hz, Ar-H), 7.81 (s, 1H, Ar-H), 8.01 (d, 2H, *J* = 8.0 Hz, Ar-H), 11.28 (brs, 1H, NH). **MS** *m/z* (%): 438.5 (8.2, M^+^).

#### (E)-N-(4-(3-(4-Chlorophenyl)acryloyl)phenyl)-2-morpholinoacetamide (24)

^1^H-NMR (CDCl_3_-d_6_) δ 2.58–2.76 (m, 4H, morpholine-H), 3.23–3.40 (m, 4H, morpholine-H), 3.85 (s, 2H, CH_2_), 7.40 (d, 2H, *J* = 8.0 Hz, Ar-H), 7.49 (s, 1H, Olefinic-H), 7.52 (s, 1H, Olefinic-H), 7.59 (d, 2H, *J* = 7.0 Hz, Ar-H), 7.74 (d, 2H, *J* = 8.5 Hz, Ar-H), 7.96 (brs, 1H, NH), 8.05 (d, 2H, *J* = 7.0 Hz, Ar-H). ^13^C-NMR δ 25.7, 26.5, 53.6, 66.6, 118.8, 118.9, 122.1, 128.5, 129.3, 129.6, 130.0, 130.8, 133.5, 133.9, 135.0, 136.4, 139.7, 141.6, 143.1, 164.2, 188.6. **MS** *m/z* (%): 384.8 (1.4, M^+^).

#### (E)-N-(4-(3-(4-Methoxyphenyl)acryloyl)phenyl)-2-morpholinoacetamide (25)

^1^H-NMR (CDCl_3_-d_6_) δ 2.64–2.72 (m, 4H, morpholine-H), 3.55–3.61 (m, 4H, morpholine-H), 3.79 (s, 2H, CH_2_), 3.89 (s, 3H, OCH_3_), 7.23 (d, 2H, *J* = 6.5 Hz, Ar-H), 7.42 (s, 1H, Olefinic-H), 7.50 (s, 1H, Olefinic-H), 7.86 (d, 2H, *J* = 7.0 Hz, Ar-H), 7.94 (d, 2H, *J* = 8.5 Hz, Ar-H), 8.15 (d, 2H, *J* = 7.5 Hz, Ar-H), 10.99 (brs, 1H, NH). ^13^C-NMR δ 23.8, 24.6, 46.7, 52.9, 55.6, 62.1, 114.2, 115.1, 117.9, 118.0, 118.8, 119.2, 122.4, 124.0, 126.7, 128.4, 133.0, 135.8, 144.0, 148.2, 156.7, 188.9. **MS** *m/z* (%): 380.4 (11.2, M^+^).

##### (E)-N-(4-(3-(3,4-Dimethoxyphenyl)acryloyl)phenyl)-2-morpholinoacetamide (26)

^1^H-NMR (CDCl_3_-d_6_) δ 2.66–2.71 (m, 4H, morpholine-H), 3.49–3.55 (m, 4H, morpholine-H), 3.67 (s, 3H, OCH_3_), 3.80 (s, 2H, CH_2_), 3.88 (s, 3H, OCH_3_), 7.42 (d, 2H, *J* = 7.0 Hz, Ar-H), 7.55 (s, 1H, Olefinic-H), 7.64 (s, 1H, Olefinic-H), 7.88 (d, 2H, *J* = 7.0 Hz, Ar-H), 7.91 (s, 1H, Ar-H), 8.10 (d, 2H, *J* = 8.5 Hz, Ar-H), 11.45 (brs, 1H, NH). ^13^C-NMR δ 15.3, 23.0, 24.6, 52.3, 55.7, 60.9, 64.7, 113.2, 114.5, 116.8, 118.0, 118.9, 122.4, 123.9, 124.4, 130.1, 133.8, 142.7, 143.6, 148.7, 152.0, 166.7, 189.2. **MS** *m/z* (%): 410.4 (32.6, M^+^).

#### (E)-2-Morpholino-N-(4-(3-(3,4,5-trimethoxyphenyl)acryloyl)phenyl)acetamide (27)

^1^H-NMR (CDCl_3_-d_6_) δ 2.34–2.86 (m, 4H, morpholine-H), 3.43–3.68 (m, 4H, morpholine-H), 3.65 (s, 3H, OCH_3_), 3.74 (s, 2H, CH_2_), 3.88 (s, 3H, OCH_3_), 3.95 (s, 3H, OCH_3_), 7.05 (s, 1H, Ar-H), 7.24 (d, 2H, *J* = 8.0 Hz, Ar-H), 7.45 (s, 1H, Olefinic-H), 7.55 (s, 1H, Ar-H), 7.60 (s, 1H, Olefinic-H), 7.94 (d, 2H, *J* = 7.0 Hz, Ar-H), 11.25 (brs, 1H, NH). ^13^C-NMR δ 14.9, 15.3, 48.7, 50.2, 55.6, 56.1, 55.9, 66.0, 112.6, 114.3, 115.9, 118.1, 118.9, 122.4, 125.7, 129.1, 133.0, 135.7, 138.4, 140.2, 144.9, 148.2, 159.7, 188.4. **MS** *m/z* (%): 440.5 (0.9, M^+^).

#### (E)-N-(4-(3-(4-Chlorophenyl)acryloyl)phenyl)-2-(4-methylpiperazin-1-yl)acetamide (28)

^1^H-NMR (CDCl_3_-d_6_) δ 1.25 (s, 3H, CH_3_), 1.52–1.55 (m, 4H, piperazine-H), 2.04 (t, 2H, piperazine-H), 2.10 (t, 2H, piperazine-H), 3.49 (s, 2H, CH_2_), 7.21 (d, 2H, *J* = 7.5 Hz, Ar-H), 7.42 (s, 1H, Olefinic-H), 7.55 (s, 1H, Olefinic-H), 7.64 (d, 2H, *J* = 8.0 Hz, Ar-H), 7.85 (d, 2H, *J* = 8.5 Hz, Ar-H), 8.25 (d, 2H, *J* = 7.0 Hz, Ar-H), 9.73 (s, 1H, NH). ^13^C-NMR δ 15.2, 25.7, 26.4, 52.9, 55.9, 67.0, 114.6, 116.7, 118.1, 119.4, 121.5, 123.0, 125.9, 126.3, 128.2, 130.4, 134.0, 139.0, 144.2, 148.6, 166.9, 186.9. **MS** *m/z* (%): 397.9 (9.5, M^+^).

#### (E)-N-(4-(3-(4-Methoxyphenyl)acryloyl)phenyl)-2-(4-methylpiperazin-1-yl)acetamide (29)

^1^H-NMR (CDCl_3_-d_6_) δ 1.14 (s, 3H, CH_3_), 1.45–1.53 (m, 4H, piperazine-H), 2.11–2.34 (m, 4H, piperazine-H), 3.40 (s, 2H, CH_2_), 3.88 (s, 3H, OCH_3_), 7.39 (s, 1H, Olefinic-H), 7.50 (d, 2H, *J* = 8.0 Hz, Ar-H), 7.62 (s, 1H, Olefinic-H), 7.78 (d, 2H, *J* = 8.0 Hz, Ar-H), 7.84 (d, 2H, *J* = 8.0 Hz, Ar-H), 8.15 (d, 2H, *J* = 7.0 Hz, Ar-H), 10.35 (s, 1H, NH). ^13^C-NMR δ 13.5, 23.8, 24.6, 49.1, 52.4, 55.8, 66.0, 112.5, 114.8, 116.0, 118.7, 120.4, 128.1, 130.9, 133.2, 134.6, 138.2, 141.5, 146.1, 148.7, 155.0, 167.8, 189.2. **MS** *m/z* (%): 393.4 (13.4, M^+^).

#### (E)-N-(4-(3-(3,4-dimethoxyphenyl)acryloyl)phenyl)-2-(4-methylpiperazin-1-yl)acetamide (30)

^1^H-NMR (CDCl_3_-d_6_) δ 1.23 (s, 3H, CH_3_), 1.21–1.43 (m, 4H, piperazine-H), 2.59–2.73 (m, 4H, piperazine-H), 3.66 (s, 2H, CH_2_), 3.88 (s, 3H, OCH_3_), 3.94 (s, 3H, OCH_3_), 7.41 (s, 1H, Olefinic-H), 7.58 (s, 1H, Ar-H), 7.70 (s, 1H, Olefinic-H), 7.78 (d, 2H, *J* = 8.5 Hz, Ar-H), 7.89 (d, 2H, *J* = 8.0 Hz, Ar-H), 8.00 (d, 2H, *J* = 6.5 Hz, Ar-H), 9.88 (s, 1H, NH). **MS** *m/z* (%): 423.5 (8.2, M^+^).

#### (E)-2-(4-Methylpiperazin-1-yl)-N-(4-(3-(3,4,5-trimethoxyphenyl)acryloyl)phenyl)acetamide (31)

^1^H-NMR (CDCl_3_-d_6_) δ 1.19 (s, 3H, CH_3_), 1.22–1.43 (m, 4H, piperazine-H), 2.54–2.72 (m, 4H, piperazine-H), 3.52 (s, 2H, CH_2_), 3.88 (s, 3H, OCH_3_), 3.94 (s, 3H, OCH_3_), 3.99 (s, 3H, OCH_3_), 7.33 (s, 1H, Olefinic-H), 7.51 (s, 1H, Ar-H), 7.65 (s, 1H, Olefinic-H), 7.72 (s, 1H, Ar-H), 7.99 (d, 2H, *J* = 8.0 Hz, Ar-H), 8.03 (d, 2H, *J* = 6.5 Hz, Ar-H), 11.36 (s, 1H, NH). ^13^C-NMR δ 15.2, 24.9, 25.3, 41.0, 45.7, 55.8, 56.4, 60.1, 66.9, 112.0, 113.9, 115.7, 117.2, 120.4, 125.8, 129.0, 131.5, 132.7, 135.8, 139.4, 144.2, 148.7, 151.7, 167.1, 188.0. **MS** *m/z* (%): 453.5 (24.6, M^+^).

#### (E)-N-(4-(3-(4-Chlorophenyl)acryloyl)phenyl)-2-(4-phenylpiperazin-1-yl)acetamide (32)

^1^H-NMR (CDCl_3_-d_6_) δ 1.39–1.50 (m, 4H, piperazine-H), 2.77–2.84 (m, 4H, piperazine-H), 3.70 (s, 2H, CH_2_), 7.41 (s, 1H, Olefinic-H), 7.58 (s, 1H, Olefinic-H), 7.72–7.86 (m, 5H, Ar-H), 7.88 (d, 2H, *J* = 8.0 Hz, Ar-H), 7.95 (d, 2H, *J* = 7.5 Hz, Ar-H), 7.95 (d, 2H, *J* = 7.5 Hz, Ar-H), 8.04 (m, 2H, Ar-H), 10.45 (s, 1H, NH). ^13^C-NMR δ 23.5, 24.1, 44.8, 52.0, 61.4, 112.4, 114.5, 117.9, 118.1, 118.9, 122.0, 123.4, 125.7, 127.1, 128.9, 129.2, 130.5, 132.8, 133.5, 137.0, 141.2, 144.1, 149.6, 157.2, 164.8, 188.0. **MS** *m/z* (%): 459.9 (7.5, M^+^).

#### (E)-N-(4-(3-(4-Methoxyphenyl)acryloyl)phenyl)-2-(4-phenylpiperazin-1-yl)acetamide (33)

^1^H-NMR (CDCl_3_-d_6_) δ 1.35–1.52 (m, 4H, piperazine-H), 2.77–2.82 (m, 4H, piperazine-H), 3.65 (s, 2H, CH_2_), 3.73 (s, 3H, OCH_3_), 7.22 (s, 1H, Olefinic-H), 7.63 (s, 1H, Olefinic-H), 7.66–7.89 (m, 5H, Ar-H), 7.90 (d, 2H, *J* = 6.0 Hz, Ar-H), 7.99 (d, 2H, *J* = 7.5 Hz, Ar-H), 8.09 (m, 4H, Ar-H), 8.12 (d, 2H, *J* = 9.0 Hz, Ar-H), 9.40 (s, 1H, NH). ^13^C-NMR δ 24.9, 25.8, 48.9, 53.1, 55.9, 65.3, 110.0, 111.9, 113.8, 115.2, 118.9, 120.0, 122.2, 124.3, 127.3, 128.4, 130.6, 131.4, 133.6, 142.9, 147.8, 148.5, 152.0, 152.4, 156.2, 166.4, 187.3. **MS** *m/z* (%): 455.5 (21.3, M^+^).

#### (E)-N-(4-(3-(3,4-dimethoxyphenyl)acryloyl)phenyl)-2-(4-phenylpiperazin-1-yl)acetamide (34)

^1^H-NMR (CDCl_3_-d_6_) δ 1.35–2.62 (m, 8H, piperazine-H), 3.54 (s, 2H, CH_2_), 3.76 (s, 3H, OCH_3_), 3.90 (s, 3H, OCH_3_), 7.34 (s, 1H, Olefinic-H), 7.55 (s, 1H, Ar-H), 7.70 (s, 1H, Olefinic-H), 7.75–7.99 (m, 5H, Ar-H), 8.01 (d, 4H, *J* = 6.0 Hz, Ar-H), 8.15 (m, 2H, Ar-H), 9.99 (s, 1H, NH). ^13^C-NMR δ 26.8, 27.1, 46.0, 53.9, 55.8, 56.9, 66.3, 111.5, 113.6, 114.9, 115.7, 118.0, 118.7, 120.4, 121.5, 122.7, 124.4, 126.8, 130.6, 131.9, 132.7, 135.0, 141.0, 144.6, 146.8, 150.7, 152.9, 162.0, 188.7. **MS** *m/z* (%): 485.5 (20.8, M^+^).

#### (E)-2-(4-Phenylpiperazin-1-yl)-N-(4-(3-(3,4,5-trimethoxyphenyl)acryloyl)phenyl)acetamide (35)

^1^H-NMR (CDCl_3_-d_6_) δ 1.35–1.68 (m, 4H, piperazine-H), 2.11–2.46 (m, 4H, piperazine-H), 3.50 (s, 2H, CH_2_), 3.64 (s, 3H, OCH_3_), 3.89 (s, 3H, OCH_3_), 4.21 (s, 3H, OCH_3_), 6.81 (s, 1H, Olefinic-H), 7.06 (s, 1H, Ar-H), 7.29 (s, 1H, Ar-H), 7.54 (s, 1H, Olefinic-H), 7.61–7.86 (m, 5H, Ar-H), 7.94 (d, 2H, *J* = 8.0 Hz, Ar-H), 8.06 (d, 2H, *J* = 8.5 Hz, Ar-H), 10.19 (s, 1H, NH). ^13^C-NMR δ 22.6, 25.7, 41.0, 46.3, 55.8, 56.3, 60.7, 62.4, 110.0, 112.7, 113.5, 115.2, 118.4, 118.7, 118.9, 120.6, 122.4, 124.6, 125.0, 127.9, 130.9, 131.4, 136.0, 141.2, 143.6, 143.9, 155.7, 161.8, 169.0, 189.7. **MS** *m/z* (%): 515.6 (10.7, M^+^).

#### (E)-N-(4-(3-(4-Chlorophenyl)acryloyl)phenyl)-3-(piperidin-1-yl)propanamide (36)

^1^H-NMR (CDCl_3_-d_6_) δ 1.24 (t, 2H, *J* = 12.0 Hz, CH_2_), 1.72–1.76 (m, 4H, piperidine-H), 2.57–2.59 (m, 4H, piperidine-H), 2.72 (t, 2H, piperazine-H), 3.72 (t, 2H, *J* = 12.5 Hz, CH_2_), 7.39 (d, 2H, *J* = 6.5 Hz, Ar-H), 7.51 (s, 1H, Olefinic-H), 7.53 (s, 1H, Olefinic-H), 7.57 (d, 2H, *J* = 8.0 Hz, Ar-H), 7.70 (d, 2H, *J* = 8.0 Hz, Ar-H), 8.08 (d, 2H, *J* = 8.0 Hz, Ar-H), 11.73 (s, 1H, NH). ^13^C-NMR δ 18.4, 24.1, 26.1, 32.6, 53.7, 54.1, 66.5, 118.8, 118.9, 120.6, 122.3, 126.0, 127.9, 129.2, 129.6, 130.1, 132.9, 133.6, 136.3, 142.7, 143.4, 171.1, 188.6. **MS** *m/z* (%): 396.9 (27.5, M^+^).

#### (E)-N-(4-(3-(4-Methoxyphenyl)acryloyl)phenyl)-3-(piperidin-1-yl)propanamide (37)

^1^H-NMR (CDCl_3_-d_6_) δ 1.65–1.68 (m, 4H, piperidine-H), 1.89–1.93 (m, 6H, piperidine-H), 3.13 (t, 2H, *J* = 1.5 Hz, CH_2_), 3.74 (s, 3H, OCH_3_), 3.85 (t, 2H, *J* = 2.0 Hz, CH_2_), 6.75 (d, 2H, *J* = 7.0 Hz, Ar-H), 6.95 (d, 2H, *J* = 13.5 Hz, Ar-H), 7.30 (s, 1H, Olefinic-H), 7.40 (s, 1H, Olefinic-H), 7.60 (d, 2H, *J* = 7.5 Hz, Ar-H), 7.78 (d, 2H, *J* = 8.0 Hz, Ar-H), 9.43 (s, 1H, NH). ^13^C-NMR δ 22.5, 22.8, 24.2, 31.8, 44.5, 47.5, 53.8, 55.3, 114.1, 114.4, 119.3, 119.5, 119.7, 127.8, 129.2, 129.6, 130.0, 130.4, 133.8, 142.7, 144.1, 161.5, 168.0, 189.2. **MS** *m/z* (%): 392.5 (6.1, M^+^).

#### (E)-3-(Piperidin-1-yl)-N-(4-(3-(3,4,5-trimethoxyphenyl)acryloyl)phenyl)propanamide (38)

^1^H-NMR (CDCl_3_-d_6_) δ 1.23 (t, 4H, *J* = 6.5 Hz, piperidine-H), 1.74–1.77 (m, 6H, piperidine-H), 2.61 (t, 2H, *J* = 5.5 Hz, CH_2_), 2.76 (t, 2H, *J* = 4.0 Hz, CH_2_), 3.72 (s, 3H, OCH_3_), 3.89 (s, 3H, OCH_3_) , 7.26 (s, 1H, Ar-H), 7.40 (s, 1H, Olefinic-H), 7.42 (s, 1H, Olefinic-H), 7.70 (d, 2H, *J* = 7.5 Hz, Ar-H), 7.98 (d, 2H, *J* = 7.0 Hz, Ar-H), 8.02 (d, 2H, *J* = 7.0 Hz, Ar-H), 11.65 (brs, 1H, NH). ^13^C-NMR δ 18.5, 23.9, 25.9, 32.6, 53.6, 54.1, 56.3, 58.4, 61.0, 105.6, 114.2, 117.9, 118.8, 118.9, 121.3, 122.6, 127.9, 130.0, 130.6, 133.2, 140.3, 143.2, 144.5, 153.5, 188.9. **MS** *m/z* (%): 422.5 (20.7, M^+^).

#### (E)-3-(Piperidin-1-yl)-N-(4-(3-(3,4,5-trimethoxyphenyl)acryloyl)phenyl)propanamide (39)

^1^H-NMR (CDCl_3_-d_6_) δ 1.20–1.80 (m, 10H, piperidine-H), 2.54 (t, 2H, *J* = 2.5 Hz, CH_2_), 2.81 (t, 2H, *J* = 2.0 Hz, CH_2_), 3.69 (s, 3H, OCH_3_), 3.89 (s, 3H, OCH_3_) , 3.92 (s, 3H, OCH_3_), 7.26 (s, 1H, Ar-H), 7.40 (s, 1H, Olefinic-H), 7.42 (s, 1H, Olefinic-H), 7.52 (s, 1H, Ar-H), 7.70 (d, 2H, *J* = 7.5 Hz, Ar-H), 8.02 (d, 2H, *J* = 9.5 Hz, Ar-H), 10.57 (brs, 1H, NH). ^13^C-NMR δ 24.1, 25.4, 28.7, 41.0, 45.7, 55.8, 57.1, 60.3, 62.7, 66.9, 110.3, 112.8, 114.0, 116.7, 118.0, 118.4, 119.5, 120.7, 122.4, 124.9, 128.7, 136.7, 144.8, 153.4, 162.7, 188.7. **MS** *m/z* (%): 452.5 (27.5, M^+^).

#### (E)-N-(4-(3-(4-Chlorophenyl)acryloyl)phenyl)-3-morpholinopropanamide (40)

^1^H-NMR (CDCl_3_-d_6_) δ 1.24–1.59 (m, 4H, morpholine-H), 2.24–2.64 (m, 4H, morpholine-H), 2.74 (t, 2H, *J* = 1.5 Hz, CH_2_), 2.79 (t, 2H, *J* = 1.5 Hz, CH_2_), 7.31 (s, 1H, Olefinic-H), 7.42 (s, 1H, Olefinic-H), 7.95 (d, 4H, *J* = 7.5 Hz, Ar-H), 8.14 (d, 4H, *J* = 9.5 Hz, Ar-H), 10.18 (brs, 1H, NH). ^13^C-NMR δ 24.5, 25.9, 41.7, 52.7, 59.1, 61.7, 110.5, 118.7, 119.2, 120.4, 122.4, 130.6, 133.2, 134.7, 136.0, 138.4, 140.5, 143.8, 149.7, 153.7, 164.7, 189.1. **MS** *m/z* (%): 398.8 (0.8, M^+^).

#### (E)-N-(4-(3-(4-Methoxyphenyl)acryloyl)phenyl)-3-morpholinopropanamide (41)

^1^H-NMR (CDCl_3_-d_6_) δ 1.29–1.44 (m, 4H, morpholine-H), 2.53–2.66 (m, 4H, morpholine-H), 2.71 (t, 2H, *J* = 1.5 Hz, CH_2_), 2.82 (t, 2H, *J* = 1.5 Hz, CH_2_), 3.88 (s, 3H, OCH_3_), 7.44 (s, 1H, Olefinic-H), 7.53 (s, 1H, Olefinic-H), 7.64 (d, 2H, *J* = 7.5 Hz, Ar-H), 7.72 (d, 2H, *J* = 8.0 Hz, Ar-H), 7.94 (d, 2H, *J* = 7.5 Hz, Ar-H), 8.04 (d, 2H, *J* = 9.5 Hz, Ar-H), 10.57 (brs, 1H, NH). ^13^C-NMR δ 23.1, 24.7, 43.8, 51.2, 55.8, 59.7, 60.0, 116.7, 118.9, 120.1, 124.0, 126.3, 127.8, 128.4, 130.9, 133.7, 135.2, 139.7, 142.8, 152.0, 158.3, 164.2, 187.2. **MS** *m/z* (%): 394.4 (12.3, M^+^).

#### (E)-N-(4-(3-(3,4-Dimethoxyphenyl)acryloyl)phenyl)-3-morpholinopropanamide (42)

^1^H-NMR (CDCl_3_-d_6_) δ 1.24 (t, 4H, *J* = 12.0 Hz, morpholine-H), 2.85–3.02 (m, 4H, morpholine-H), 3.72 (t, 2H, *J* = 2.5 Hz, CH_2_), 3.79 (t, 2H, *J* = 2.0 Hz, CH_2_), 3.93 (s, 3H, OCH_3_), 3.96 (s, 3H, OCH_3_), 7.16 (d, 2H, *J* = 7.0 Hz, Ar-H), 7.22 (d, 2H, *J* = 7.5 Hz, Ar-H), 7.37 (s, 1H, Olefinic-H), 7.40 (s, 1H, Olefinic-H), 7.73 (brs, 1H, NH), 7.77 (s, 1H, Ar-H), 8.02 (d, 2H, *J* = 7.0 Hz, Ar-H). ^13^C-NMR δ 32.1, 52.7, 53.9, 56.0, 56.1, 65.9, 110.1, 111.1, 114.6, 115.2, 116.8, 118.9, 119.0, 119.7, 123.1, 127.9, 129.9, 133.8, 142.4, 144.7, 149.2, 151.4, 166.7, 189.0. **MS** *m/z* (%): 424.5 (10.1, M^+^).

#### (E)-3-Morpholino-N-(4-(3-(3,4,5-trimethoxyphenyl)acryloyl)phenyl)propanamide (43)

^1^H-NMR (CDCl_3_-d_6_) δ 1.22–1.24 (m, 4H, morpholine-H), 1.25–1.27 (m, 4H, morpholine-H), 2.75 (t, 2H, *J* = 2.0 Hz, CH_2_), 2.89 (t, 2H, *J* = 2.5 Hz, CH_2_), 3.81 (s, 3H, OCH_3_), 3.90 (s, 3H, OCH_3_), 3.92 (s, 3H, OCH_3_), 6.86 (s, 1H, Ar-H), 7.03 (s, 1H, Ar-H), 7.39 (d, 2H, *J* = 8.5 Hz, Ar-H), 7.44 (s, 1H, Olefinic-H), 7.53 (s, 1H, Olefinic-H), 8.00 (d, 2H, *J* = 7.0 Hz, Ar-H), 10.99 (brs, 1H, NH). ^13^C-NMR δ 18.5, 32.2, 52.8, 53.9, 56.0, 61.0, 66.5, 105.6, 106.9, 110.5, 112.9, 113.4, 117.0, 118.9, 119.0, 121.2, 130.1, 130.5, 133.6, 134.4, 142.7, 144.6, 153.5, 167.9, 188.9. **MS** *m/z* (%): 454.5 (13.4, M^+^).

#### (E)-N-(4-(3-(4-Chlorophenyl)acryloyl)phenyl)-3-(4-methylpiperazin-1-yl)propanamide (44)

^1^H-NMR (CDCl_3_-d_6_) δ 1.15 (s, 3H, CH_3_), 1.20–1.29 (m, 4H, piperazine-H), 1.76–1.95 (m, 4H, piperazine-H), 2.54 (t, 2H, *J* = 1.5 Hz, CH_2_), 2.79 (t, 2H, *J* = 2.0 Hz, CH_2_), 7.24 (d, 2H, *J* = 8.5 Hz, Ar-H), 7.24 (s, 1H, Olefinic-H), 7.42 (s, 1H, Olefinic-H), 7.61 (d, 4H, *J* = 8.5 Hz, Ar-H), 8.14 (d, 2H, *J* = 7.5 Hz, Ar-H), 11.63 (brs, 1H, NH). ^13^C-NMR δ 15.2, 23.4, 25.0, 41.3, 49.8, 57.8, 64.9, 112.8, 113.9, 115.0, 118.9, 121.7, 124.0, 127.3, 128.4, 133.9, 136.5, 140.7, 141.2, 144.9, 150.7, 162.4, 186.7. **MS** *m/z* (%): 411.9 (22.5, M^+^).

#### (E)-N-(4-(3-(4-Methoxyphenyl)acryloyl)phenyl)-3-(4-methylpiperazin-1-yl)propanamide (45)

^1^H-NMR (CDCl_3_-d_6_) δ 1.11 (s, 3H, CH_3_), 1.25–1.34 (m, 4H, piperazine-H), 1.52–1.71 (m, 4H, piperazine-H), 2.63 (t, 2H, *J* = 2.5 Hz, CH_2_), 2.84 (t, 2H, *J* = 2.0 Hz, CH_2_), 3.87 (s, 3H, OCH_3_), 5.34 (s, 1H, NH), 7.21 (s, 1H, Olefinic-H), 7.33 (d, 4H, *J* = 8.0 Hz, Ar-H), 7.45 (s, 1H, Olefinic-H), 7.94 (d, 4H, *J* = 8.0 Hz, Ar-H). ^13^C-NMR δ 14.0, 24.2, 26.1, 39.2, 42.8, 55.9, 57.8, 60.4, 112.4, 116.8, 119.4, 121.1, 123.5, 125.0, 127.4, 128.0, 128.9, 133.4, 135.7, 138.7, 141.6, 155.4, 160.9, 189.2. **MS** *m/z* (%): 407.5 (1.9, M^+^).

#### (E)-N-(4-(3-(3,4-Dimethoxyphenyl)acryloyl)phenyl)-3-(4-methylpiperazin-1-yl)propanamide (46)

^1^H-NMR (CDCl_3_-d_6_) δ 1.20 (s, 3H, CH_3_), 1.24–1.36 (m, 4H, piperazine-H), 1.47–1.76 (m, 4H, piperazine-H), 2.68 (t, 2H, *J* = 1.5 Hz, CH_2_), 2.89 (t, 2H, *J* = 2.5 Hz, CH_2_), 3.87 (s, 3H, OCH_3_), 3.94 (s, 3H, OCH_3_), 6.41 (s, 1H, NH), 7.36 (s, 1H, Olefinic-H), 7.49 (d, 2H, *J* = 6.5 Hz, Ar-H), 7.53 (s, 1H, Ar-H), 7.55 (s, 1H, Olefinic-H), 8.00 (d, 4H, *J* = 8.0 Hz, Ar-H). **MS** *m/z* (%): 437.5 (29.4, M^+^).

#### (E)-3-(4-Methylpiperazin-1-yl)-N-(4-(3-(3,4,5-trimethoxyphenyl)acryloyl)phenyl)propanamide (47)

^1^H-NMR (CDCl_3_-d_6_) δ 1.17 (s, 3H, CH_3_), 1.24–1.76 (m, 8H, piperazine-H), 3.01 (t, 2H, *J* = 2.5 Hz, CH_2_), 3.22 (t, 2H, *J* = 2.0 Hz, CH_2_), 3.89 (s, 3H, OCH_3_), 3.94 (s, 3H, OCH_3_), 4.15 (s, 3H, OCH_3_), 7.40 (s, 1H, Olefinic-H), 7.55 (s, 1H, Ar-H), 7.59 (d, 4H, *J* = 8.5 Hz, Ar-H), 7.60 (s, 1H, Ar-H), 7.67 (s, 1H, Olefinic-H), 8.41 (s, 1H, NH). ^13^C-NMR δ 11.9, 21.8, 26.7, 37.5, 41.0, 55.8, 56.4, 59.1, 60.7, 64.3, 110.4, 118.7, 118.9, 119.4, 120.7, 123.5, 127.4, 126.8, 129.0, 131.4, 132.7, 146.2, 148.1, 152.0, 164.7, 182.0. **MS** *m/z* (%): 467.5 (17.0, M^+^).

#### (E)-N-(4-(3-(4-Chlorophenyl)acryloyl)phenyl)-3-(4-phenylpiperazin-1-yl)propanamide (48)

^1^H-NMR (CDCl_3_-d_6_) δ 1.24–1.46 (m, 4H, piperazine-H), 1.72–1.99 (m, 4H, piperazine-H), 2.87 (t, 2H, *J* = 2.5 Hz, CH_2_), 3.13 (t, 2H, *J* = 1.0 Hz, CH_2_), 7.23 (s, 1H, Olefinic-H), 7.44 (s, 1H, Olefinic-H), 7.52 (d, 2H, *J* = 8.5 Hz, Ar-H), 7.60 (d, 2H, *J* = 8.0 Hz, Ar-H), 7.84 (d, 4H, *J* = 8.5 Hz, Ar-H), 7.91–8.05 (m, 5H, Ar-H), 10.13 (s, 1H, NH). ^13^C-NMR δ 24.2, 27.5, 41.1, 50.3, 57.8, 60.1, 110.3, 113.6, 115.2, 118.0, 118.9, 120.8, 122.5, 125.1, 127.1, 130.6, 133.7, 137.2, 139.4, 140.2, 143.6, 144.8, 148.6, 150.2, 155.9, 159,0, 164.3, 187.5. **MS** *m/z* (%): 473.1 (12.8, M^+^).

#### (E)-N-(4-(3-(4-Methoxyphenyl)acryloyl)phenyl)-3-(4-phenylpiperazin-1-yl)propanamide (49)

^1^H-NMR (CDCl_3_-d_6_) δ 1.27–1.40 (m, 4H, piperazine-H), 1.69–1.83 (m, 4H, piperazine-H), 3.88 (s, 3H, OCH_3_), 2.90 (t, 2H, *J* = 2.0 Hz, CH_2_), 2.95 (t, 2H, *J* = 1.0 Hz, CH_2_), 6.82 (s, 1H, Olefinic-H), 7.49 (s, 1H, Olefinic-H), 7.60 (d, 2H, *J* = 7.5 Hz, Ar-H), 7.71–7.79 (m, 5H, Ar-H), 7.81 (d, 2H, *J* = 8.0 Hz, Ar-H), 7.89 (d, 2H, *J* = 8.0 Hz, Ar-H), 7.96 (d, 2H, *J* = 8.0 Hz, Ar-H), 11.34 (s, 1H, NH). ^13^C-NMR δ 21.4, 23.6, 41.7, 49.7, 55.9, 60.1, 65.3, 112.4, 115.1, 115.4, 118.1, 119.3, 121.4, 122.0, 125.8, 127.3, 134.6, 139.2, 141.7, 142.5, 144.3, 147.4, 148.0, 150.6, 152.3, 155.2, 158.2, 163.4, 189.4. **MS** *m/z* (%): 469.5 (11.0, M^+^).

#### (E)-N-(4-(3-(3,4-Dimethoxyphenyl)acryloyl)phenyl)-3-(4-phenylpiperazin-1-yl)propanamide (50)

^1^H-NMR (CDCl_3_-d_6_) δ 1.27–1.75 (m, 8H, piperazine-H), 3.85 (s, 3H, OCH_3_), 3.89 (s, 3H, OCH_3_), 2.79 (t, 2H, *J* = 2.5 Hz, CH_2_), 3.01 (t, 2H, *J* = 1.5 Hz, CH_2_), 7.19 (s, 1H, Olefinic-H), 7.40 (s, 1H, Olefinic-H), 7.55 (d, 2H, *J* = 8.0 Hz, Ar-H), 7.63 (s, 1H, Ar-H), 7.75–7.99 (m, 5H, Ar-H), 8.01 (d, 2H, *J* = 8.0 Hz, Ar-H), 8.17 (d, 2H, *J* = 8.5 Hz, Ar-H), 10.27 (s, 1H, NH). ^13^C-NMR δ 23.4, 25.1, 41.7, 44.5, 55.8, 56.4, 59.1, 66.0, 110.7, 115.9, 117.3, 118.0, 118.9, 121.5, 122.4, 123.8, 125.0, 125.7, 127.9, 129.3, 130.2, 133.7, 139.0, 141.2, 143.6, 147.2, 159.4, 167.2, 189.4. **MS** *m/z* (%): 499.6 (32.5, M^+^).

#### (E)-3-(4-Phenylpiperazin-1-yl)-N-(4-(3-(3,4,5-trimethoxyphenyl)acryloyl)phenyl)propanamide (51)

^1^H-NMR (CDCl_3_-d_6_) δ 1.30–1.64 (m, 8H, piperazine-H), 3.85 (s, 3H, OCH_3_), 3.89 (s, 3H, OCH_3_), 4.01 (s, 3H, OCH_3_), 2.61 (t, 2H, CH_2_), 2.96 (t, 2H, CH_2_), 7.28 (s, 1H, Olefinic-H), 7.42 (s, 1H, Olefinic-H), 7.54 (s, 1H, Ar-H), 7.64 (s, 1H, Ar-H), 7.69 (d, 2H, *J* = 7.5 Hz, Ar-H), 7.73–8.06 (m, 5H, Ar-H), 8.11 (d, 2H, *J* = 8.5 Hz, Ar-H), 11.03 (s, 1H, NH). ^13^C-NMR δ 24.1, 26.8, 43.8, 54.9, 55.8, 56.1, 59.3, 60.7, 64.3, 110.1, 112.5, 116.1, 117.3, 118.9, 121.0, 122.6, 125.5, 126.1, 126.8, 127.6, 129.4, 131.0, 133.7, 135.4, 139.7, 141.2, 143.7, 150.7, 166.9, 184.2. **MS** *m/z* (%): 529.6 (29.1, M^+^).

### Determination of *in vitro* antimicrobial activity

The primary screen was carried out using the agar disc-diffusion method^27^ using Müller–Hinton agar medium. Sterile filter paper discs (8 mm diameter) were moistened with the compound solution in dimethylsulfoxide of specific concentration 200 µg/disc, the antibacterial antibiotic ampicillin, ciprofloxacin, and the antifungal drug Clotrimazole (100 µg/disc), used as positive control, were carefully placed on the agar cultures plates that had been previously inoculated separately with the microorganisms. The plates were incubated at 37 °C, and the clear zone (inhibition zone) around each compound was measured in (mm) the diameter after 24 h in case of bacteria and at 25 °C for 48 h in case of fungi The Minimal inhibitory concentrations (MIC) and the minimal bactericidal concentrations (MBC) for the compounds **36**, **37**, **38**, **42**, and **44** against the same microorganisms used in the primary screening were carried out using the microdilution susceptibility method in Müller–Hinton Broth^28^ The same compounds except **42** and **44** were tested for bio-film activity against *Staphylococcus aureus* IFO 3060, *Micrococcus luteus* IFO 3232 and *Pseudomonas aeuroginosa* IFO 3448 The tested compounds and antimicrobial standard solution were dissolved in dimethylsulfoxide at concentration of 64 µg/ml. The twofold dilutions of the solution were prepared (64, 32, …, 0.5 µg/ml). The microorganism suspensions at 10^6^ CFU/ml (colony forming unit/ml) concentrations were inoculated to the corresponding wells. The plates were incubated at 37 °C for 24 h. The MIC values were determined as the lowest concentration that inhibited the growth of the microorganism and the MBC values were determined by the lowest concentration that killed of the microorganism by re-cultured on solid medium to verify the absence of growth The anti-biofilm activity was done as follows, two-fold serial dilutions of tested compounds were made in sterile 96-well tissue culture plates containing 50 µl of Mueller–Hinton broth per well. A 50 µl of fresh bacterial suspension (1.0 McFarland) was added to each well. Positive control (bacterial cells + broth) and negative control were included. After incubation at 37 °C for 48 h, the biofilm biomass was assayed using the crystal violet staining assay method^29^. The biofilm inhibition concentration was defined as the lowest concentration of the tested compound that showed 50% inhibition on the biofilm formation.

### Molecular modelling

The three-dimensional structures of some selected substituted amide chalcone derivatives, which represent best anti-biofilm inhibitor, in their neutral forms, were built by using the MOE of Chemical Computing Group Inc. software (Montreal, Canada). The Lowest energy conformer of new analogues “global-minima” was docked into the binding pocket of Cyclic di-GMP (c-di-GMP) that is a widely conserved second-messenger Synthesis of c-di-GMP occurs via diguanylate cyclase (DGC) enzymes encoding of c-di-GMP occurs via phosphodiesterase (PDE) enzymes. DGCs is of fundamental importance for c-di-GMP signalling and cellular homeostasis^30^. It was obtained from the Protein Data Bank of Brookhaven National Laboratory. The hydrogens were added, then enzyme structure was subjected a refinement protocol where the constraints on the enzyme were gradually removed and minimised until the RMSD gradient was 0.01 kcal/mol Å. Energy minimisation was carried out using the molecular mechanics force field “AMBER”. For each quinazoline derivative, energy minimisations (EM) were performed using 1000 steps of steepest descent, followed by conjugate gradient minimisation to a RMSD energy gradient of 0.01 kcal/mol Å. The active site of the enzyme was detected using a radius of 10.0 Å around MTX. The energy of binding was calculated as the difference between the energy of the complex and individual energies of the enzyme and ligand^31–34^.

The compounds under study underwent flexible alignment experiment using “Molecular Operating Environment” software (MOE of Chemical Computing Group Inc., on a Core i7 2.3 GHz workstation, Montreal, Canada). The molecules were constructed using the Builder module of MOE. Their geometry was optimised by using the MMFF94 forcefield followed by a flexible alignment using systematic conformational search. The lowest energy aligned conformers were identified. ADMET Calculations were determined using implemented tool in MOE, 2009.10.

## Results and discussion

### Chemistry

**Scheme 1.**
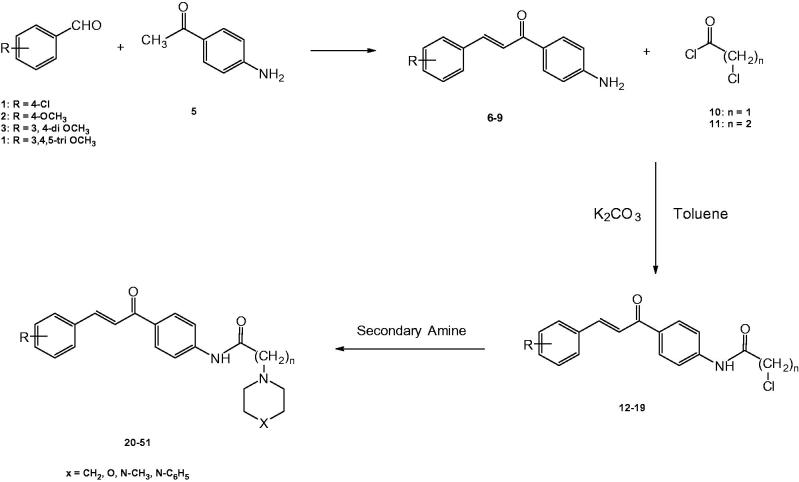
Synthesis of the target compounds **20**–**51**.

The synthetic strategy to prepare the new target compounds was outlined in Scheme 1. The amino function of 1-(4-aminophenyl)-3-(substituted phenyl)prop-2-en-1-one analogues **6**–**9** was acylated with either 2-chloroacetyl chloride (**10**) or 3-chloropropionyl chloride (**11**) in presence of potassium carbonate in dry toluene to yield (*E*)-2-chloro-*N*-(4-(3-(substituedphenyl)acryloyl)phenyl)acetamides **(12**–**15)**, (*E*)-3-chloro-*N*-(4-(3-(substitutedphenyl)acryloyl)phenyl)propanamides **(16**–**19)**. The ^1^H-NMR spectra of the synthesised intermediates (**12**–**15**) proved to accommodate the –COCH_2_Cl moiety into the structures of **6**–**9** by the appearance of singlet integrated for two protons at a range of δ 4.28–4.56 ppm depending on the type of substituent on the phenyl ring. The integration of –COCH_2_CH_2_Cl moiety to produce the intermediates **16**–**19** was also proved by the appearance of a set of two triplets integrated for four protons at a range of δ 2.92–3.91 ppm. The target compounds **20**–**51**, were obtained by the reaction of the intermediate derivatives **12**–**19** with a variety of secondary amines in dry toluene (Scheme 1, Table 1). ^1^H-NMR spectra proved the inclusion of the secondary amines into the structures of the target compounds. Piperidine appeared as either two sets of triplets multiplets at δ 1.58 and 2.84 or as one multiplet in the range of δ 1.20–3.18 integrated as ten protons; morpholine appeared as two multiplets at δ 1.24, 3.86 ppm integrated for eight protons; piperazines showed either sets of triplets and multiplets at δ 1.52 and 2.10 or two multiplets at δ 1.21–2.84 ppm integrated for eight protons, ^13^C-NMR and Mass spectral analyses confirmed the aforementioned findings.

### *In vitro* antimicrobial activity

The synthesised compounds were tested for their *in vitro* antimicrobial activity against a panel of standard strains of the *Gram-positive* bacteria (*Staphylococcus aureus* IFO 3060, *Bacillus subtilis* IFO 3007, and *Micrococcus luteus* IFO 3232), the *Gram-negative* bacteria (*Escherichia coli* IFO 3301 and *Pseudomonas aeuroginosa* IFO 3448), and the pathogenic fungus *Candida albicans* IFO 0583, *Aspergillus oryzae* IFO 4177, and *Aspergillus niger* IFO 4414. The primary screen was carried out using the agar disc-diffusion method^27^. The results of the preliminary antimicrobial screening of the synthesised compounds are shown in Table 2. The results revealed that the majority of the synthesised compounds showed varying degrees of inhibition against the tested microorganisms. *Gram-positive* bacteria are considered the most sensitive among the tested microorganisms. Compounds **36**, **37**, **38**, **42**, and **44** showed good activity against *Gram-positive* and fungi. The MIC and MBC for the most active compounds **36**, **37**, **38**, **42**, and **44** against the same microorganism used in the primary screening were carried out using the microdilution susceptibility method in Müller–Hinton Broth^28^ as shown in Table 3. Compounds **36**, **37**, and **38** show good MIC and MBC against tested organisms. The compounds **36**, **37**, and **38** show anti-biofilm activity (IC_50_, µg/ml) against *Staphylococcus aureus* IFO 3060, *Micrococcus luteus* IFO 3232, and *Pseudomonas aeuroginosa* IFO 3448. Compound **36** shows the most potent anti-biofilm activity among all tested compounds as shown in Table 4 using the crystal violet staining assay method^31^.

**Table 2.** Antimicrobial activity of tested compounds (200 μg/8 mm disc) against *Gram positive bacteria* (*Staphylococcus aureus* IFO 3060, *Bacillus subtilis* IFO 3007, *Micrococcus luteus* IFO 3232), *Gram negative bacteria* (*Escherichia coli* IFO 3301, *Pseudomonas aeuroginosa* IFO 3448), and Fungi (*Candida albicans* IFO 0583, *Aspergillus oryzae* IFO 4177 and *Aspergillus niger* IFO 4414).

Compound No.	Diameter of inhibition zone (mm)							
	*Gram positive bacteria*			*Gram positive bacteria*		Fungi		
	*S. aureus*	*B. subtilis*	*M. luteus*	*E. coli*	*P. aeuroginosa*	*C. albicans*	*A. oryzae*	*A. niger*
**20**	–	14	11	12	10	–	–	–
**21**	9	12	10	14	–	10	–	10
**22**	–	14	–	–	–	11	–	–
**23**	–	13	12	11	–	10	9	–
**24**	10	–	–	13	9	13	–	9
**25**	–	–	12	14	13	–	–	–
**26**	–	12	–	–	–	–	10	–
**27**	14	16	15	12	–	–	–	–
**28**	12	14	–	–	–	12	–	–
**29**	14	16	–	–	–	–	–	–
**30**	14	16	12	–	–	–	–	–
**31**	18	20	16	14	10	–	–	–
**32**	–	15	–	–	–	–	–	–
**33**	10	12	10	–	–	–	–	–
**34**	–	13	–	11	9	–	–	–
**35**	–	11	–	10	–	–	–	–
**36**	28	29	24	18	14	20	18	14
**37**	20	26	22	19	16	21	19	20
**38**	26	28	21	15	12	20	18	17
**39**	16	18	14	–	–	–	–	–
**40**	12	12	–	11	–	10	–	–
**41**	–	14	–	12	–	–	–	–
**42**	18	20	16	14	12	18	14	12
**43**	–	–	–	–	–	–	–	–
**44**	21	22	20	18	16	12	10	–
**45**	10	12	–	14	10	10	–	–
**46**	12	15	10	–	–	–	–	–
**47**	–	12	–	10	9	11	–	–
**48**	–	14	–	–	–	–	10	11
**49**	10	15	10	12	–	12	–	–
**50**	–	11	–	–	–	–	–	–
**51**	9	12	–	9	9	9	–	–
**Ampicillin**	28	30	25	24	22	NT	NT	NT
**Ciprofloxacin**	34	38	32	38	36	NT	NT	NT
**Clotrimazole**	NT	NT	NT	NT	NT	21	22	24

– : Not active (8 mm), Weak activity (8–12 mm), Moderate activity (12–15 mm), Strong activity (>15 mm). Solvent: DMSo (8 mm).

**Table 3.** The Minimal Inhibitory Concentrations (MIC, µg/ml) and Minimal Bactericidal Concentrations (MBC µg/ml) of compounds **36**, **37**, **38**, **42**, and **44** in comparison with the broad spectrum antibacterial drug, Ampicillin or Ciprofloxacin, and antifungal drug Clotrimazole against tested microorganism.

Compound No.	*S. aureus*	*B. subtilis*	*M. luteus*	*E. coli*	*P. aeuroginosa*	*C. albicans*	*A. oryzae*	*A. niger*
**36**								
MIC	2.0	2.0	4.0	4.0	ND	2.0	4.0	5.0
MBC	4.0	6.0	8.0	8.0	ND	6.0	8.0	10.0
**37**								
MIC	3.0	4.0	4.0	2.0	4.0	3.0	4.0	6.0
MBC	6.0	8.0	9.0	4.0	8.0	6.0	8.0	10.0
**38**								
MIC	2.0	2.0	4.0	ND	ND	3.0	4.0	ND
MBC	6.0	6.0	7.0	ND	ND	6.0	10	ND
**42**								
MIC	8.0	6.0	6.0	ND	ND	4.0	ND	ND
MBC	ND	ND	ND	ND	ND	8.0	ND	ND
**44**								
MIC	4.0	4.0	6.0	4.0	ND	ND	ND	ND
MBC	8.0	8.0	ND	8.0	ND	ND	ND	ND
**Ampicillin**								
MIC	2.0	1.0	2.0	1.0	ND	ND	ND	ND
MBC	4.0	3.0	4.0	3.0	ND	ND	ND	ND
**Ciprofloxacin**								
MIC	0.5	0.5	0.5	0.25	1.0	ND	ND	ND
MBC	1.5	1.0	2.0	1.0	3.0	ND	ND	ND
**Clotrimazole**								
MIC	ND	ND	ND	ND	ND	2.0	3.0	5.0
MBC	ND	ND	ND	ND	ND	5.0	6.0	8.0

ND: not determined.

**Table 4.** Anti bio-film activity (IC_50_, µg/ml) of compounds **36, 37**, and **38** against *Staphylococcus aureus* IFO 3060, *Micrococcus luteus* IFO 3232, and *Pseudomonas aeuroginosa* IFO 3448.

Compound No.	*S. aureus*	*M. luteus*	*P. aeuroginosa*
**36**	2.4 ± 0.10	4.8 ± 0.11	7.8 ± 0.24
**37**	4.9 ± 0.21	5.7 ± 0.26	8.6 ± 0.22
**38**	2.9 ± 0.16	5.6 ± 0.22	6.4 ± 0.23
**Erythromycin**	0.45 ± 0.15	0.62 ± 0.11	0.84 ± 0.21

Results are mean values from at least three experiments ± SD.

### Structure–activity correlation

Structure–activity correlation, based on the different strains used in the biological screening, revealed that, in general, the propanamide analogues are more active than the acetamide ones **20–35** that proved to be inactive against most of the tested organisms. Some of the acetamide analogues showed moderate activity against gram-positive organisms such as compounds **30** and **31**. These compounds are characterised by having multiple methoxy groups in addition to the methyl piperazine moiety that proved to be essential for activity. On the other hand, some of the propanamide derivatives proved active against most of the tested organisms, compounds **36**, **37**, **38**, **42**, and **44** are active against gram-positive bacteria, gram-negative bacteria and even the pathogenic fungi. It was revealed that the presence of piperidine moiety favours the activity as in the case of compounds **36**, **37**, and **38** more than the morpholine moiety in **40** or *N*-methyl piperazine group in **44**. Furthermore, it was observed that the presence of phenylpiperazine moiety did not favour the activity as none of the compounds containing such group showed any kind of activity against the tested organisms.

### Molecular modelling study

#### Molecular docking study

It is becoming evident that c-di-GMP represents a pivotal second messenger which is involved in bacterial virulence-related phenotypes. Therefore, targeting the enzymes involved in c-diGMP synthesis represents an appealing strategy for the development of anti-biofilm drugs. The active site of the DGC domain of PleD from *C. crescentus* (PDB accession number 2V0N) was used for *in silico* in-depth study^30^. The definition of the I-site binding pocket provides an entry point into unravelling the molecular mechanisms of ligand–protein interactions involved in c-di-GMP signalling and makes DGCs a valuable target for drug design to develop new strategies against biofilm-related diseases. Detailed modelling study was carried out to assist the interpretation of the present data and provide information on binding induced mobility, atomistically. The molecular mechanism of product inhibition through I-site binding was quite interesting to be concerned.

**Figure 1.**
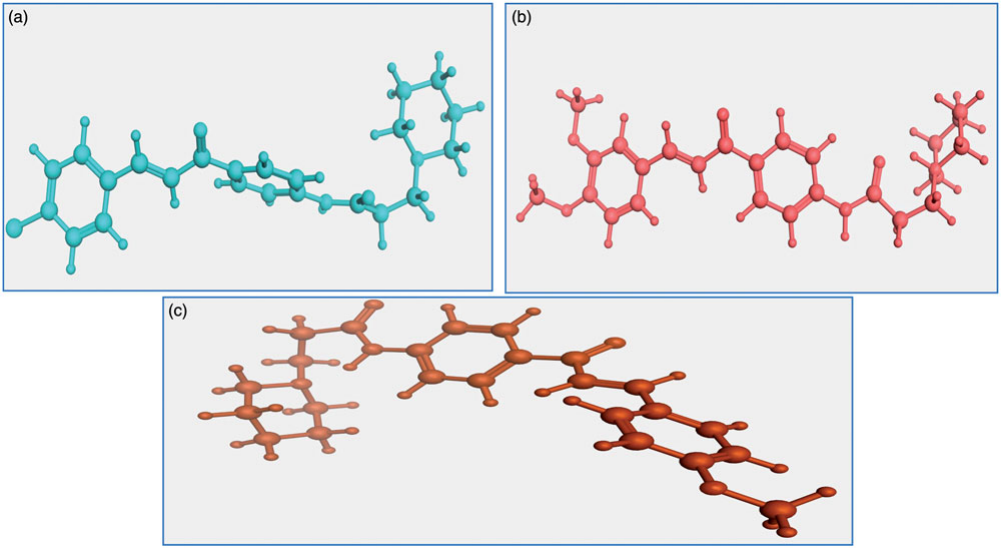
Lowest energy conformers of compound (a) **36**, (b) **38**, and (c) **37** with balls and cylinders.

**Figure 2.**
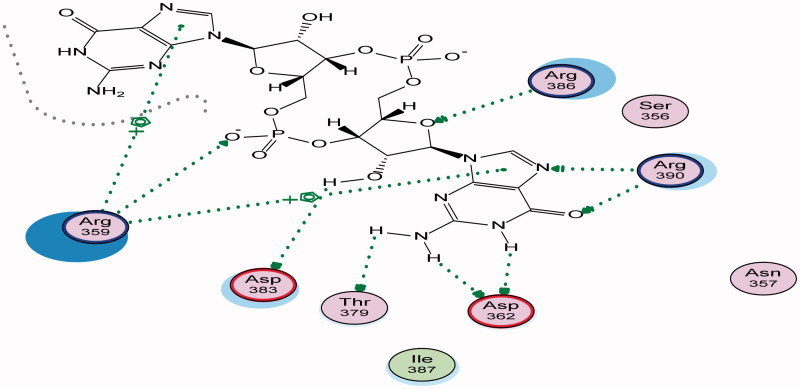
2D binding mode and residues involved in the recognition of reference ligand at active site (c-di-GMP) Arg 386, Arg 390, Asp 362 Thr 379, Asp 383, and Arg 359 via hydrogen bonding interaction.

**Figure 3.**
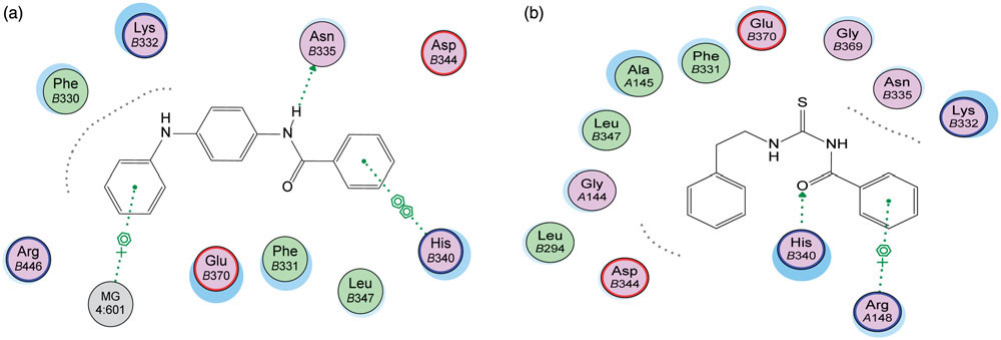
2D binding mode and residues involved in the recognition of active biofilm inhibitors (a) Compound **A**, (b) Compound **B**, ligands as references at active site (c-di-GMP).

**Figure 4.**
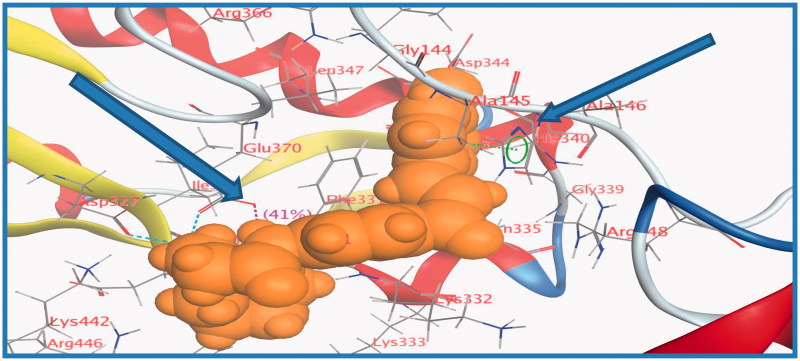
3D binding mode and residues involved in the recognition of active compound **36** at active site (c-di-GMP).

**Figure 5.**
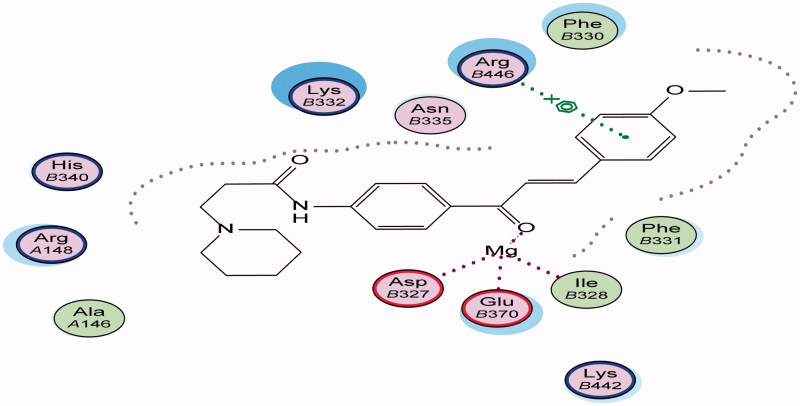
3D binding mode and residues involved in the recognition of active compound **37** at active site (c-di-GMP).

**Figure 6.**
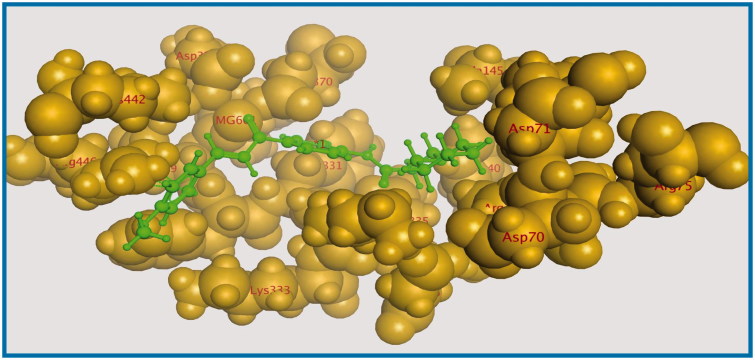
The aligned conformation of compound **37** (ball and stick) occupying pocket (space filled).

**Figure 7.**
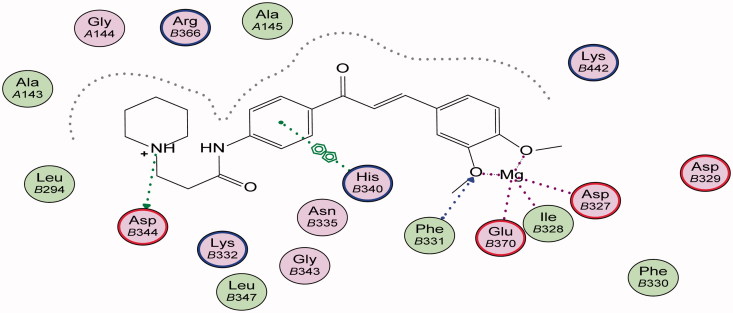
3D binding mode and residues involved in the recognition of active compound **38** at active site (c-di-GMP).

**Figure 8.**
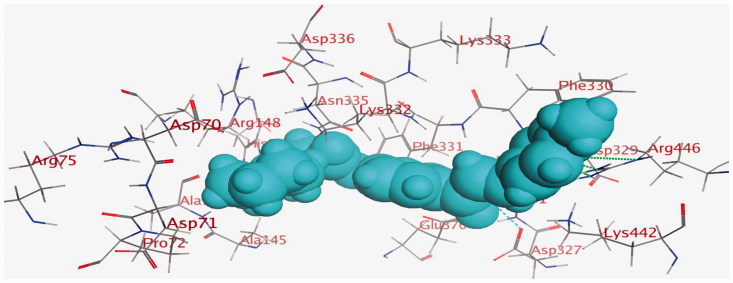
The aligned conformation of compound **38** (space filled cyan) occupying pocket c-di-GMP.

Energy minimisation and conformational search study were conducted to compounds under focus represented by **36**, **37**, and **38** (Figure 1). Docking of anti-biofilm exhibiting derivatives in addition to co-crystallised ligand PleD were used to analyse the structural transitions that occur during I-site binding of c-di-GMP. The reference ligand within the active site has been docked along with the anti-biofilm compounds under focus **36**, **37**, and **38**. A close-up view of ligand binding pocket docked reference showed binding to Arg386, Arg390, Asp362, Thr379, Asp383, and Arg359 via hydrogen bonding interaction (Figure 2). Additional small molecules that previously identified as potent DCG inhibitors were further docked into enzyme along with our compounds to validate our results^35^. Compound **A** inhibitor binds through HisB340 amino acid residue via arene–arene interaction, AsnB335 (hydrogen bonding) and Mg metal atom by amino acid network GluB370, IleB328, AspB327 (Figure 3(a)). Compound **B** inhibitor binds with HisB340 (hydrogen bonding) and Arg A148 (cationic–arene interaction) (Figure 3(b)). Three-dimensional structural complementarities between the protein binding site and the ligands represent one of the important factors determining the binding affinity. Compound **36** HisB340 (arene–arene interaction), Mg…GluB370 hydrogen bonding, IleB328, AspB327 (Figure 4). Compound **37** showed binding via amino acid residues Arg B446 (cationic–arene) in addition a triplet network binding with Mg via carbonyl oxygen through GluB370, IleB328, AspB327 (Figure 5). Compound **38** stick to active site via HisB340 (arene–arene interaction) Asp B344 (hydrogen bonding), Phe B331 (hydrogen bonding), furthermore the connection with Mg via the two methoxy groups oxygen atoms with GluB370, IleB328, AspB327 (Figure 7). Three-dimensional structural complementarities between the protein binding site and the ligands represent one of the important factors determining the binding affinity. In addition, all the tested compounds were bound in the deep cleft of the binding site that was in agreement with the binding location (Figures 4, 6, and 8). This information gave us an understanding of how the compounds act as biofilm inhibitors. General observation of docking results illustrated that the tested chalcone derivatives exhibited preponderant affinity (based on the data of binding affinity). The binding energy of the synthesised compounds showing least energy indicating higher binding to the active site, they showed less energy even than reference compounds A and B (Table 5).

**Table 5.** Docking score of compounds under study.

Compound	Dock score
**36**	−10.75799
**37**	−10.61655
**38**	−13.1454
**A**	−11.27966
**B**	−9.757301

#### Surface mapping

**Figure 9.**
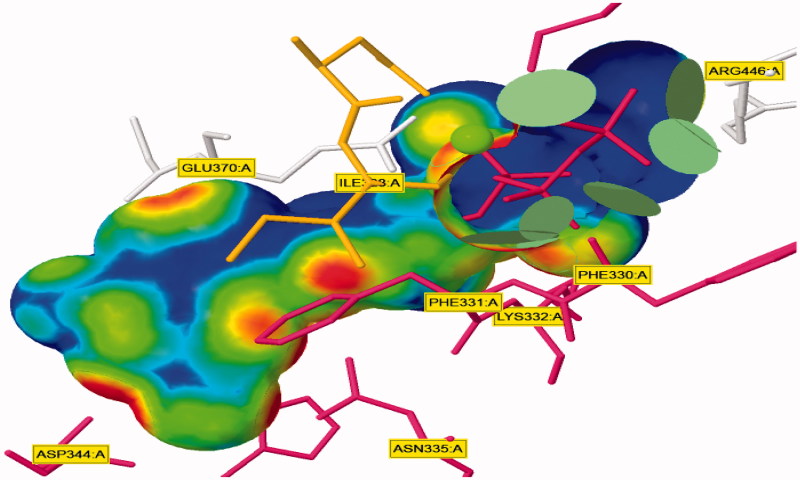
Close-up view of ligand binding pocket crystal structure of the response regulator, within a bound dimer of c-di-GMP which make specific contacts to the ligand in the crystal structure.

**Figure 10.**
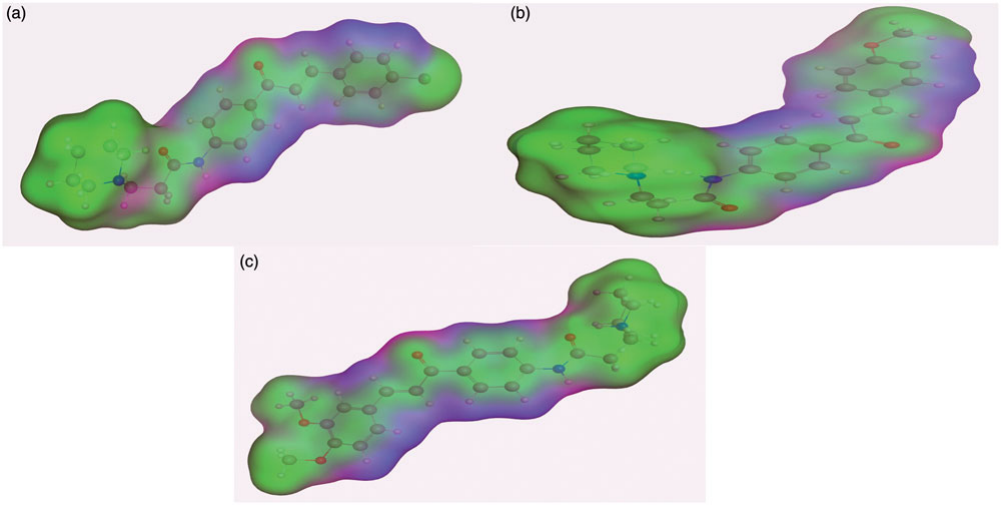
Surface map for (a) compound **36**, (b) compound **37** and (c) compound **38**, Pink, hydrogen bond, blue: mild polar, green hydrophobic.

The influence of the substituent group’s physicochemical properties on the activity of the compounds was observed. More specifically, hydrophobicity was found to be directly related to the antimicrobial activity, in agreement with other studies carried out for a different series of nifuroxazide analogues^36–38^. A surface map for the active site within c-di-GMP was exported from protein data bank site emphasising about hydrophobicity of the active site (Figure 9). Further investigations were conducted to explore the reasons behind the anti-biofilm formation activity of compounds **36**, **37**, and **38**. Hydrophobic surface mapping study revealed that compounds bearing methoxy and chloro groups showed more lipophilic character (greener regions) and hence were able to achieve more contact with the lipophobic pocket of the enzyme (Figure 10). Moreover, **35** possess a more flat structure which allows the compound to adopt conformation that utilizes more hydrophobic space inside the pocket. The obtained hydrophobic mapping and conformations emphasize a distinct role of hydrophobicity in the potential protein-binding site.

#### ADME calculations

Oral bioavailability represents essential role in the production of bioactive molecules into therapeutic agents^39^. So, it was of great importance to conduct a computational study for the prediction of ADMET properties of compounds **36**, **37**, and **38** for the determination of topological polar surface area (TPSA), and the “rule of five” formulated by Lipinski^40^ for the activity prediction of an drug-likeness and oral administered drug, if it has no more than one violation in its rules. The calculated descriptors were obtained using the MOE package, and the results are listed in Table 6. The obtained results revealed that the Log *P* are less than 5.0 for compounds **36**, **37**, and **38**, also the molecular weight was less than 500, hydrogen bond acceptor <10 and hydrogen bond donors <5 which fulfil Lipinski’s rule, number of rotatable bonds less than 10, so all the compounds fulfil Lipinski rule with zero violations except for compound **36** with one violation. In addition, the calculated total polar surface area of all compounds is considered a key property linked to drug bioavailability; the passively absorbed molecules with TPSA >140 have low oral bioavailability, all the tested compounds showed lower TPSA suggesting that compounds they can be used as good orally absorbed anti-biofilm agents with diminished toxicity among the investigated compounds.

**Table 6.** Pharmacokinetic parameters of compounds **36**, **37**, and **38**.

Compound	Mwt	TPSA	Log *P*	Lip. don	Lip. acc	Lip. V	b.rotN
**36**	396.918	49.41	4.761	1	4	1	8
**37**	392.499	58.64	4.125	1	5	0	9
**38**	423.533	69.07	3.867	2	6	0	10

TPSA: Polar surface area; Log *P*: Calculated lipophilicity; Lip. don: Number of hydrogen bond donors; Lip. acc: Number of hydrogen bond acceptors; Lip. V: Number of violations of Lipinski rule; b.rotN: No. of rotatable bond.

## Conclusions

The work reported herein provides an insight into the development of novel antimicrobial and anti-biofilm agents effective over wide range pathogenic strains. We have introduced a new class of chalcone/amine hybrid structure employing an efficient simple protocol which was evaluated for both antimicrobial and anti-biofilm activity. All the compounds showed good to moderate inhibitory and bactericidal effects over most of the *Gram-positive* and *Gram-negative* bacterial strains, respectively. Furthermore, compounds **36**, **37**, and **38** were found to be most active against biofilm formation. Their lower cytotoxicities reflect their therapeutic potential for their growth in the field of antimicrobial agents. This study contributes to the emerging understanding of the c-di-GMP regulatory network in bacteria. The current emphasis lies on the identification of some inhibitory molecules, regulatory mechanisms, with the long-term goal in mind of approaching a detailed systems-level understanding of c-di-GMP signalling. Our experiments provide an entry key point into the anti-biofilm mechanism of action using *in silico* studies such as docking, surface mapping and ADME studies.

## Supplementary Material

IENZ_1461855_Supplementary_material.pdf

## References

[1] Ugale V, Patel H, Patel B, Bari S. Benzofurano-isatins: search for antimicrobial agents. Arab J Chem 2017;10:S389–S96.

[2] Zhang X, Khalidi O, Kim SY, et al Synthesis and biological evaluation of 5,7-dihydroxyflavanone derivatives as antimicrobial agents. Bioorg Med Chem Lett 2016;26:3089–92. 27210435 10.1016/j.bmcl.2016.05.003 PMC7927313

[3] Sambanthamoorthy K, Neiditch MB, Sloup RE, et al Identification of small molecules that antagonize diguanylate cyclase enzymes to inhibit Biofilm formation. Antimicrob Agents Chemother 2012;56:5202–11. 22850508 10.1128/AAC.01396-12 PMC3457405

[4] Hall-Stoodley L, Costerton JW, Stoodley P Bacterial biofilms: from the natural environment to infectious diseases. Nat Rev Microbiol 2004; 2:95–108. 15040259 10.1038/nrmicro821

[5] Hall-Stoodley L, Stoodley P. Evolving concepts in biofilm infections. Cell Microbiol 2009;11:1034–43. 19374653 10.1111/j.1462-5822.2009.01323.x

[6] (a) Cotter PA, Stibitz S. c-di-GMP-mediated regulation of virulence and biofilm formation. Curr Opin Microbiol 2007;10:17–23. (b) De N, Pirruccello M, Krasteva PV, et al Phosphorylation-independent regulation of the diguanylate cyclase. WspR PLoS Biol 2008;6:67. 10.1016/j.mib.2006.12.006 17208514

[7] Dow JM, Fouhy Y, Lucey JF, Ryan RP. The HD-GYP domain, cyclic di-GMP signaling, and bacterial virulence to plants. Mol Plant Microbe Interact 2006;19:1378–84. 17153922 10.1094/MPMI-19-1378

[8] Sambanthamoorthy K, et al Identification of a novel benzimidazole that inhibits bacterial biofilm formation in a broad-spectrum manner. Antimicrob Agents Chemother 2011;55:4369–78. 21709104 10.1128/AAC.00583-11 PMC3165298

[9] Sambanthamoorthy K, Schwartz A, Nagarajan V, Elasri MO. The role of msa in *Staphylococcus aureus* biofilm formation. BMC Microbiol 2008;8:221. 19087289 10.1186/1471-2180-8-221 PMC2648981

[10] Schleheck D, Barraud N, Klebensberger J, et al *Pseudomonas aeruginosa* PAO1 preferentially grows as aggregates in liquid batch cultures and disperses upon starvation. PLoS One 2009;4:e5513. 19436737 10.1371/journal.pone.0005513 PMC2677461

[11] Sintim HO, Smith JA, Wang J, et al Paradigm shift in discovering next-generation anti-infective agents: targeting quorum sensing, c-di-GMP signaling and biofilm formation in bacteria with small molecules. Future Med Chem 2010;2:1005–35. 21426116 10.4155/fmc.10.185

[12] Suwito H, Ni’matuzahroh AN, Kristanti S, et al Antimicrobial activities and in silico analysis of methoxy amino chalcone derivatives. Proc Chem 2016;18:103–11.

[13] Kant R, Kumar D, Agarwal D, et al Synthesis of newer 1,2,3-triazole linked chalcone and flavone hybrid compounds and evaluation of their antimicrobial and cytotoxic activities. Eur J Med Chem 2016;113:34–49. 26922227 10.1016/j.ejmech.2016.02.041

[14] Liaras K, Geronikaki A, Glamočlija J, et al Thiazole-based chalcones as potent antimicrobial agents. Synthesis and biological evaluation. Bioorg Med Chem 2011;19:3135–40. 21524583 10.1016/j.bmc.2011.04.007

[15] Pedrosa O, Cruz D, da Viana O, et al Hybrid compounds as direct multitarget ligands: a review. Curr Top Med Chem 2017;17:1044–79. 27697048 10.2174/1568026616666160927160620

[16] Kuppast B, Fahmy H. Thiazolo[4,5-*d*]pyrimidines as a privileged scaffold in drug discovery. Eur J Med Chem 2016;113:198–213. 26942627 10.1016/j.ejmech.2016.02.031

[17] Suwitoa H, Matuzahrohb N, Kristantia AN, et al, Antimicrobial activities and in silico analysis of methoxy amino chalcone derivatives. Proc Chem 2016;18:103–11.

[18] Suwito H, Ul Haq K, Rahmah NND, et al 4-({4-[(2E)-3-(2,5-Dimethoxyphenyl)prop-2enoyl]phenyl}amino)-4-oxobutanoic acid. Molbank 2017;2017:M938.

[19] Suwito H, Jumina M, Puspaningsih P. Anticancer and antimicrobial activity of methoxy amino chalcone derivatives. Pharm Chem 2015;7:89–94.

[20] Suwito H, Kristanti AN, Hayati S, et al Antimicrobial activities and in silico analysis of methoxy amino chalcone derivatives. Proc Chem 2016;18:103–11.

[21] Hassan GS, El-Messery SM, Abbas A. Synthesis and anticancer activity of new thiazolo[3,2-a]pyrimidines: DNA binding and molecular modeling study. Bioorg Chem 2017;74:41–52. 28750204 10.1016/j.bioorg.2017.07.008

[22] El-Gazzar YI, Georgey HH, El-Messery SM, et al Synthesis, biological evaluation and molecular modeling study of new (1,2,4-triazole or 1,3,4-thiadiazole)-methylthio-derivatives of quinazolin-4(*3H*)-one as DHFR inhibitors. Bioorg Chem 2017;72:282–92. 28499189 10.1016/j.bioorg.2017.04.019

[23] El-Subbagh HI, El-Azab AS, Hassan GS, et al Thiadiazolodiazepine analogues as a new class of neuromuscular blocking agents: synthesis, biological evaluation and molecular modeling study. Eur J Med Chem 2017;126:15–23. 27744183 10.1016/j.ejmech.2016.09.096

[24] El-Subbagh HI, Hassan GS, El-Taher KEH, et al Synthesis, biological evaluation and molecular modeling study of thiadiazolo[3,2-*a*][1,3]diazepine analogues of HIE-124 as a new class of short acting hypnotics. Eur J Med Chem 2016;124:237–47. 27597405 10.1016/j.ejmech.2016.08.038

[25] Siddiqui ZN, Praveen S, Musthafa MTN, et al Thermal solvent-free synthesis of chromonyl chalcones, pyrazolines and their i*nvitro* antibacterial, antifungal activities. J Enzyme Inhibit Med Chem 2012;27:84. 10.3109/14756366.2011.577035 21612378

[26] Siddiqui ZN, Musthafa MTN, Ahmad A, Khan AU. Thermal solvent-free synthesis of novel pyrazolylchalcones and pyrazolines as potential antimicrobial agents. Bioorg Med Chem Lett 2011;21:2860. 21507638 10.1016/j.bmcl.2011.03.080

[27] Wayne PA, Clinical and Laboratory Standards Institute (CLSI). “Performance standards for antimicrobial susceptibility testing, 25th informational supplement”. 2015;M100:S15.

[28] Murray PR, Baron EJ, Pfaller MA, et al Manual of clinical microbiology. Washington DC: ASM Press; 1995.

[29] Stepanovic S, Vukovic D, Dakic I, et al A modified microtiter-plate test for quantification of staphylococcal biofilm formation. J Microbiol. Methods 2000;40:175–9. 10699673 10.1016/s0167-7012(00)00122-6

[30] Wassmann P, Chan C, Paul R, et al Structure of Bef3–modified response regulator pled: implications for diguanylate cyclase activation, catalysis, and feedback inhibition. Structure 2007;15:915. 17697997 10.1016/j.str.2007.06.016

[31] Profeta S, Allinger NL. Molecular mechanics calculations on aliphatic amines. J Am Chem Soc 1985;107:1907–18.

[32] Allinger NL. Conformational analysis. 130. MM2. A hydrocarbon force field utilizing V_1_ and V_2_ torsional terms. J Am Chem Soc 1977;99:8127–34.

[33] Labute P, Williams C, Feher M, et al Flexible alignment of small molecules. J Med Chem 2001;44:1483–90. 11334559 10.1021/jm0002634

[34] Kearsley S, Smith GM. An alternative method for the alignment of molecular structures: maximizing electrostatic and steric overlap. Tetrahedron Comput Methodol 1990;3:615–33.

[35] Sambanthamoorthy K, Neiditch MB, Sloup RE, et al Identification of small molecules that antagonize diguanylate cyclase enzymes to inhibit biofilm formation. Antimicrob Agents Chemother 2012;56:5202–11. 22850508 10.1128/AAC.01396-12 PMC3457405

[36] Tavares LC, Chisté JJ, Santos MGB, Penna TCV. Synthesis and biological activity of nifuroxazide and analogs II. Boll Chim Farm 1999;138:432. 10622109

[37] Masunari A, Tavares LC. A new class of nifuroxazide analogues: synthesis of 5-nitrothiophene derivatives with antimicrobial activity against multidrug-resistant *Staphylococcus aureus* . Bioorg Med Chem 2007;15:4229–36. 17419064 10.1016/j.bmc.2007.03.068

[38] Tavares LC, Penna TCV, Amaral AT. Synthesis and biological activity of nifuroxazide and analogs. Boll Chim Farm 1997;136:244–9. 9164164

[39] Zhao Y, Abraham MH, Lee J, et al Rate-limited steps of human oral absorption and QSAR studies. Pharm Res 2002;19: 1446–56. 12425461 10.1023/a:1020444330011

[40] Lipinski CA, Lombardo F, Dominy BW, Feeney PJ. Experimental and computational approaches to estimate solubility and permeability in drug discovery and development settings. Adv Drug Deliv Rev 1997;23:3–25. 10.1016/s0169-409x(00)00129-0 11259830

