# Supplementary figures and images for: Synthesis, antimicrobial, anti-biofilm evaluation, and molecular modelling study of new chalcone linked amines derivatives
Source: J Enzyme Inhib Med Chem. 2018 May 3;33(1):818–32. doi: 10.1080/14756366.2018.1461855 (PMC6009874; doi:10.1080/14756366.2018.1461855)

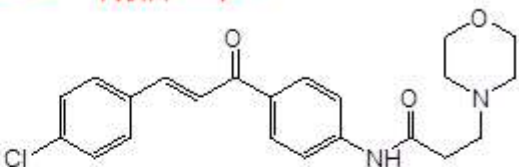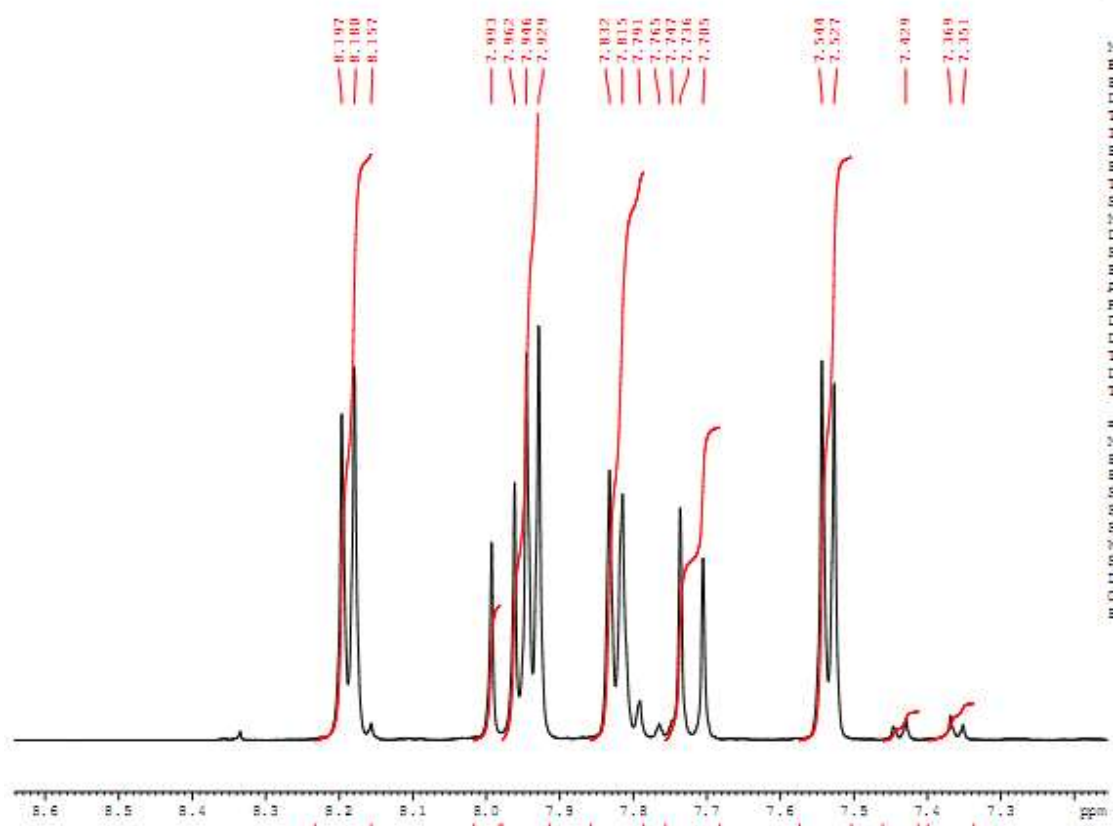

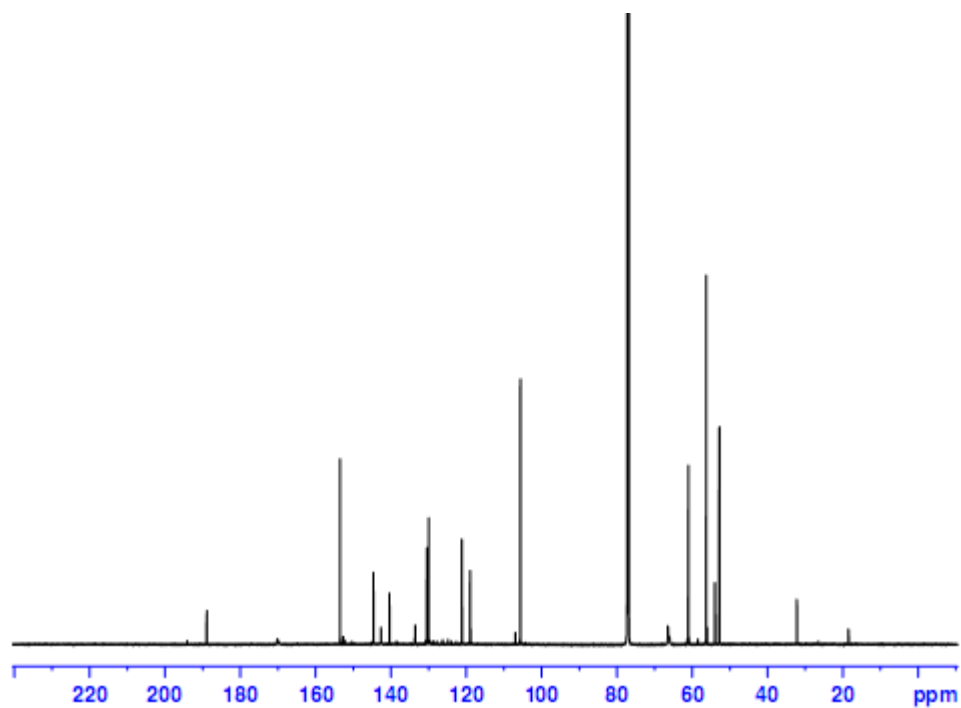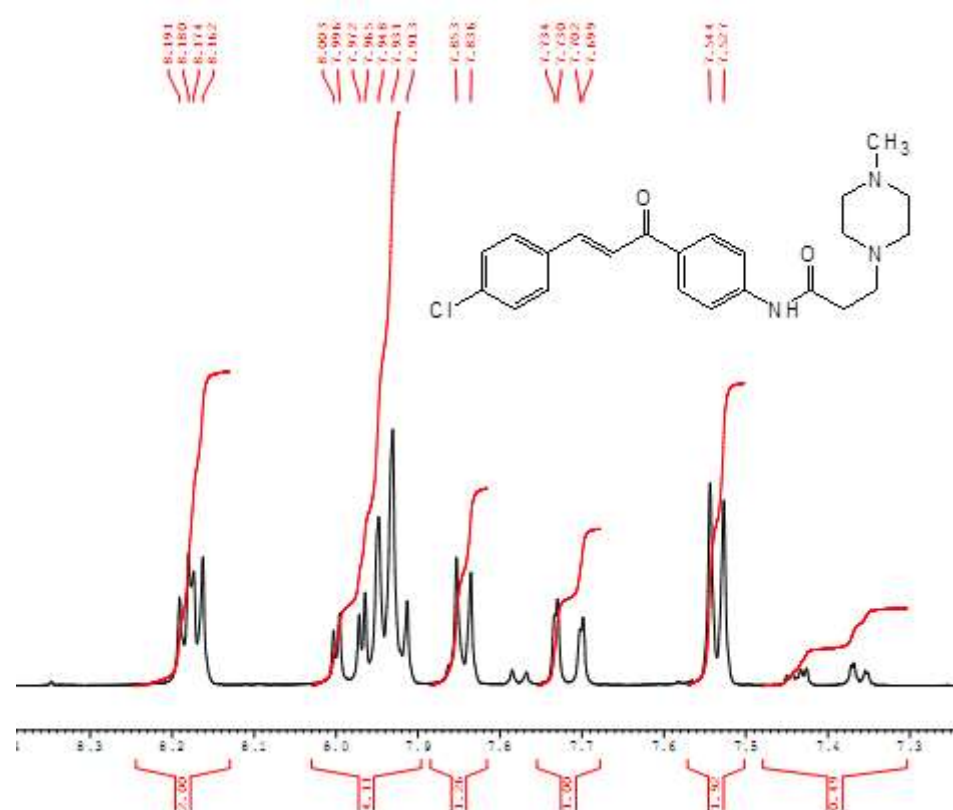

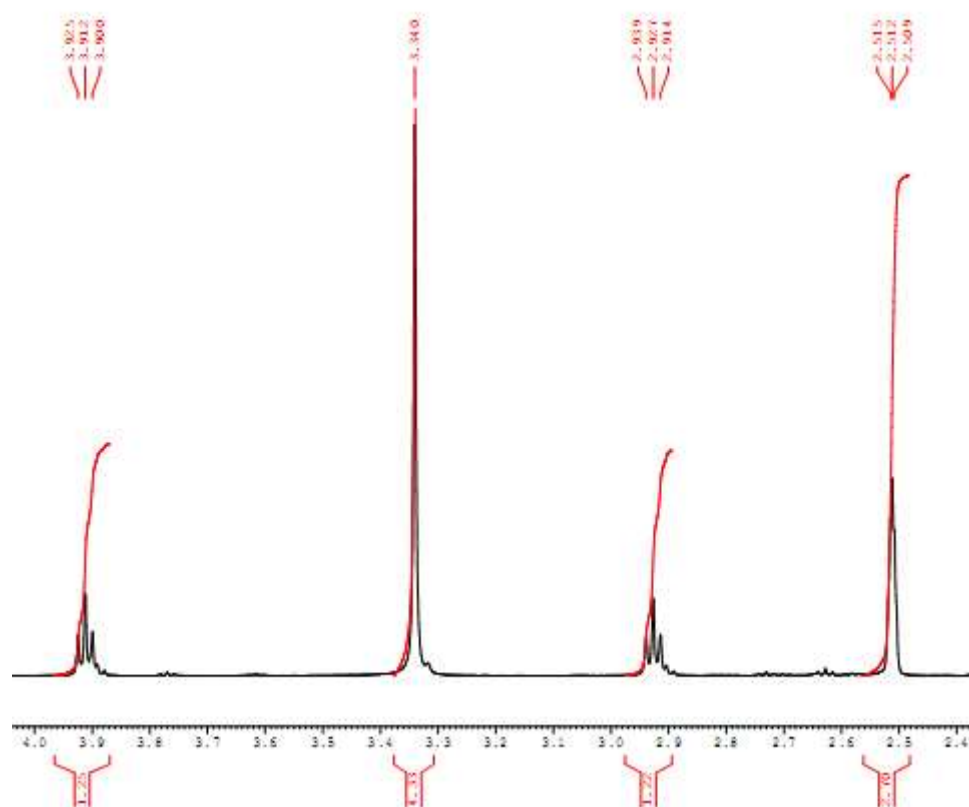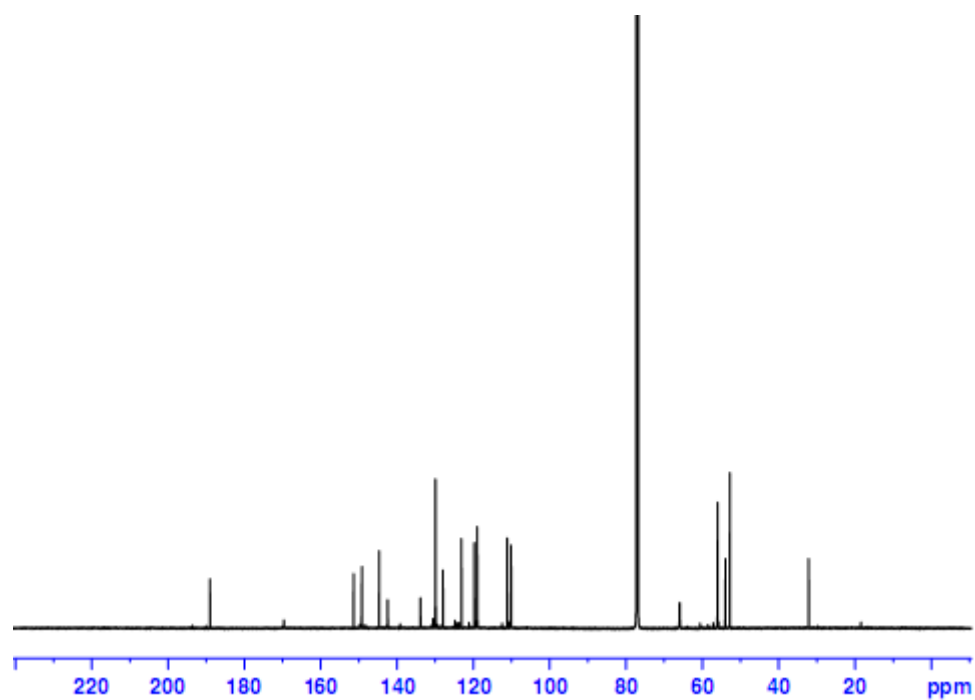

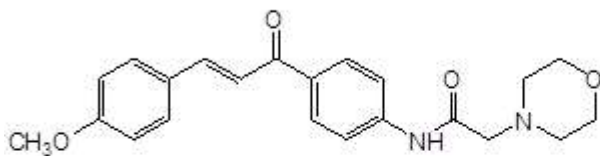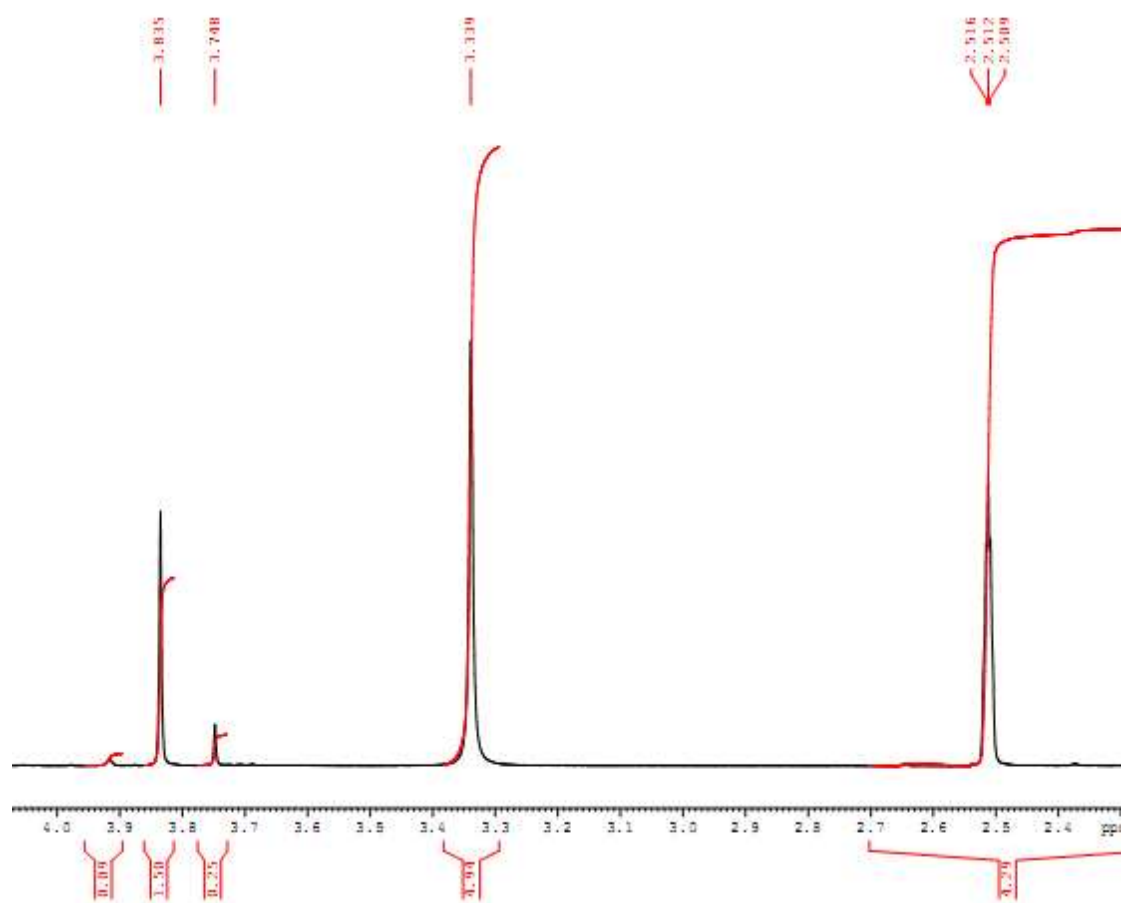

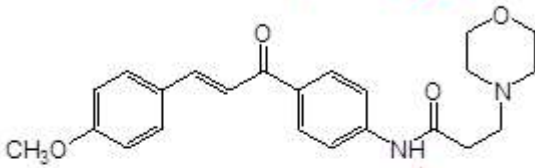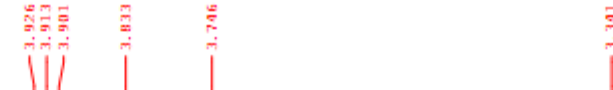

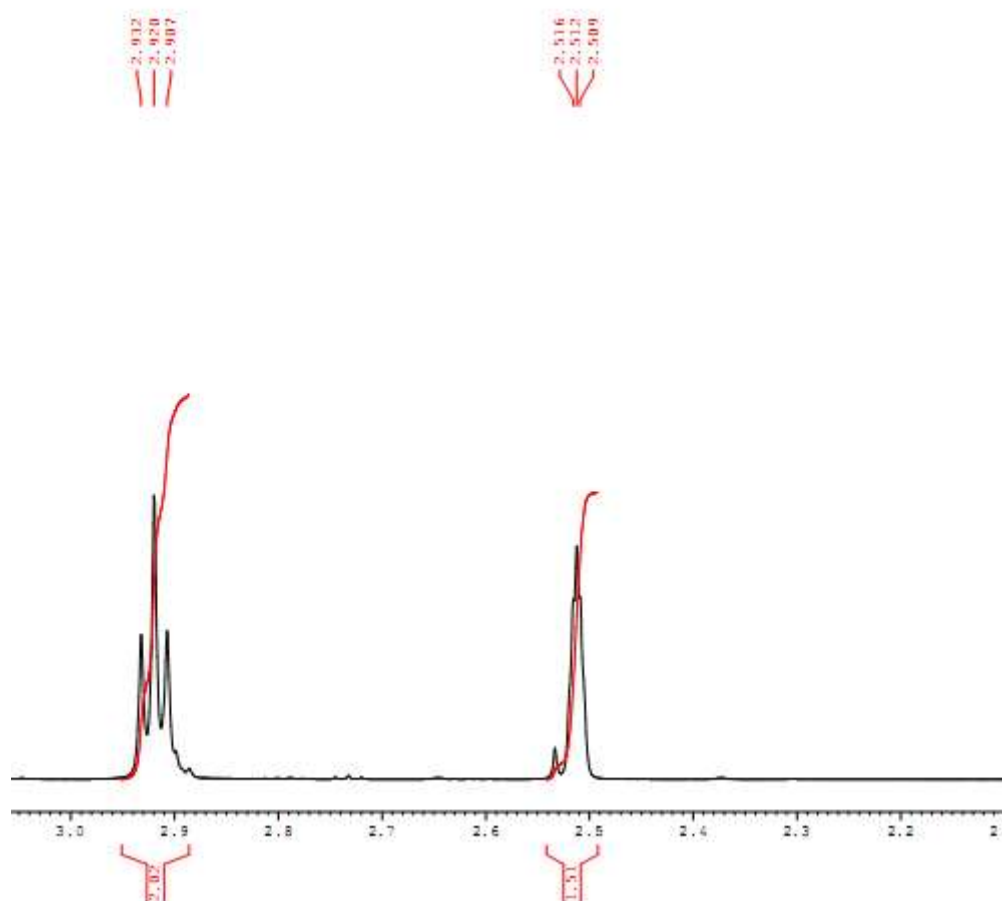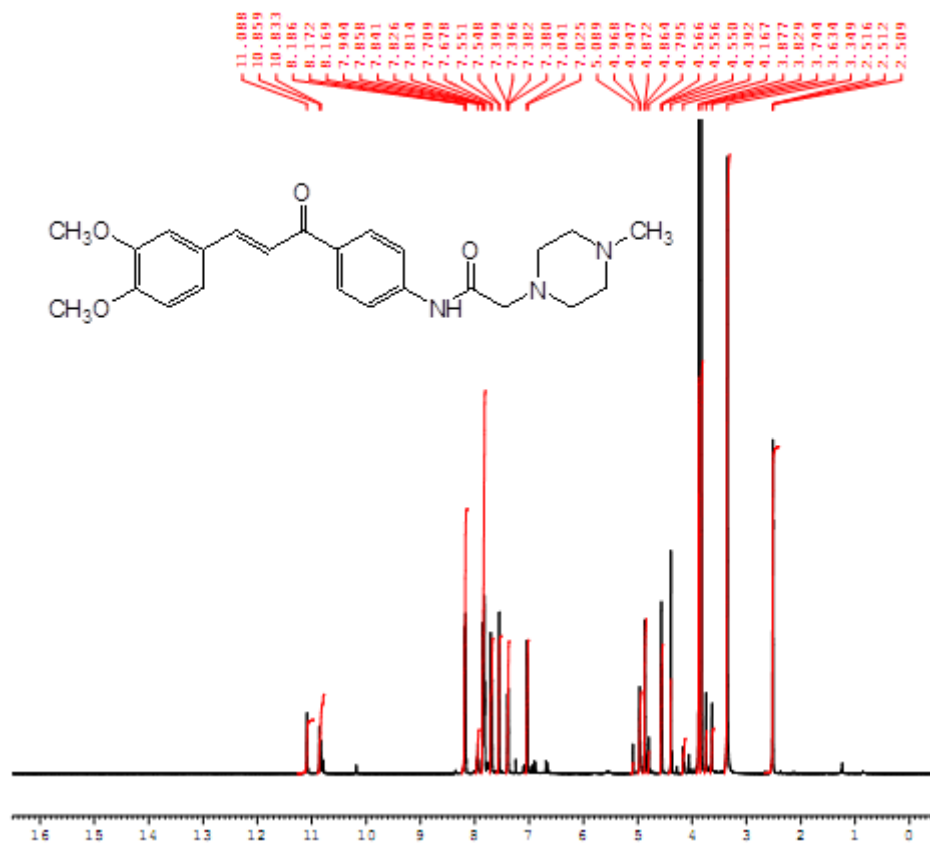

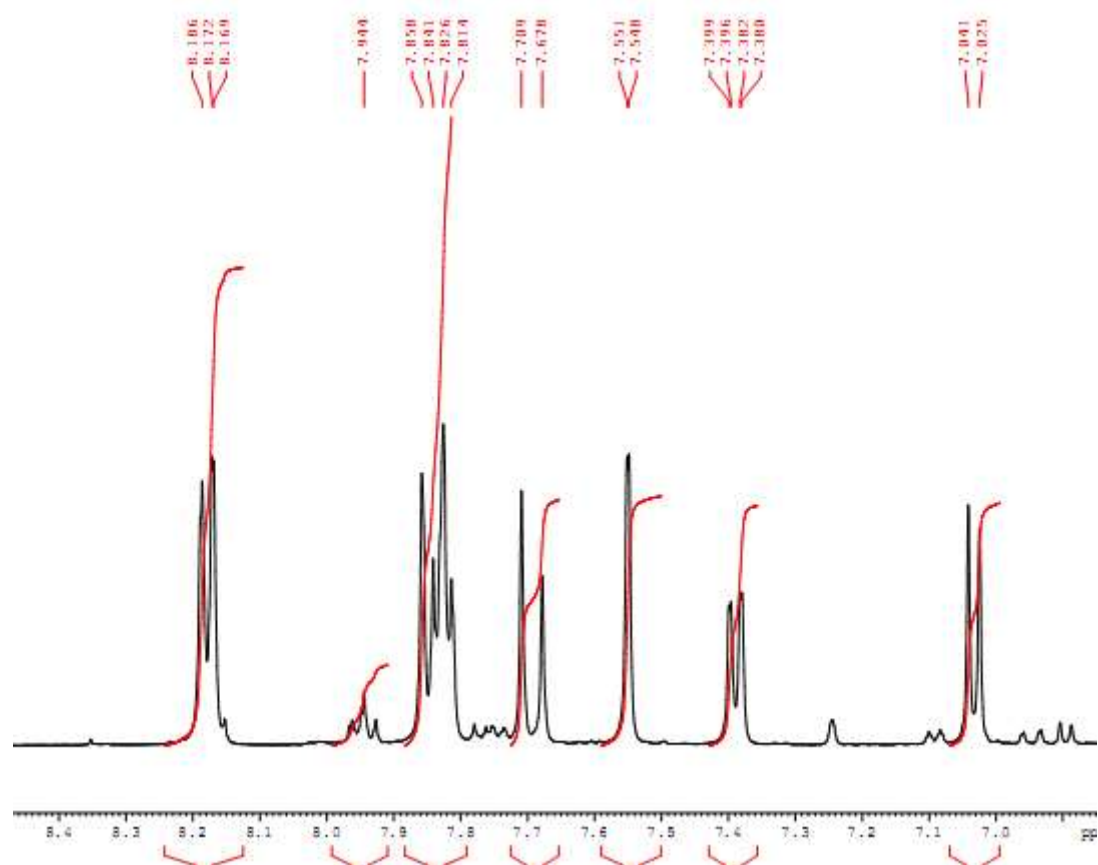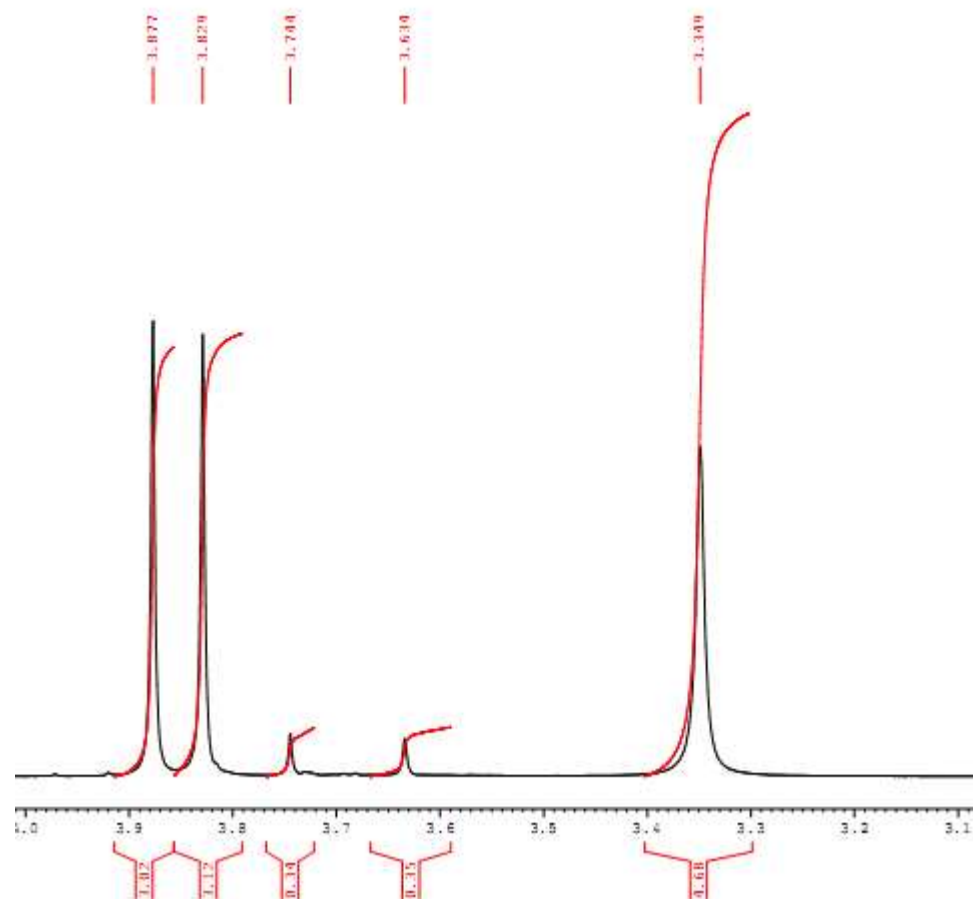

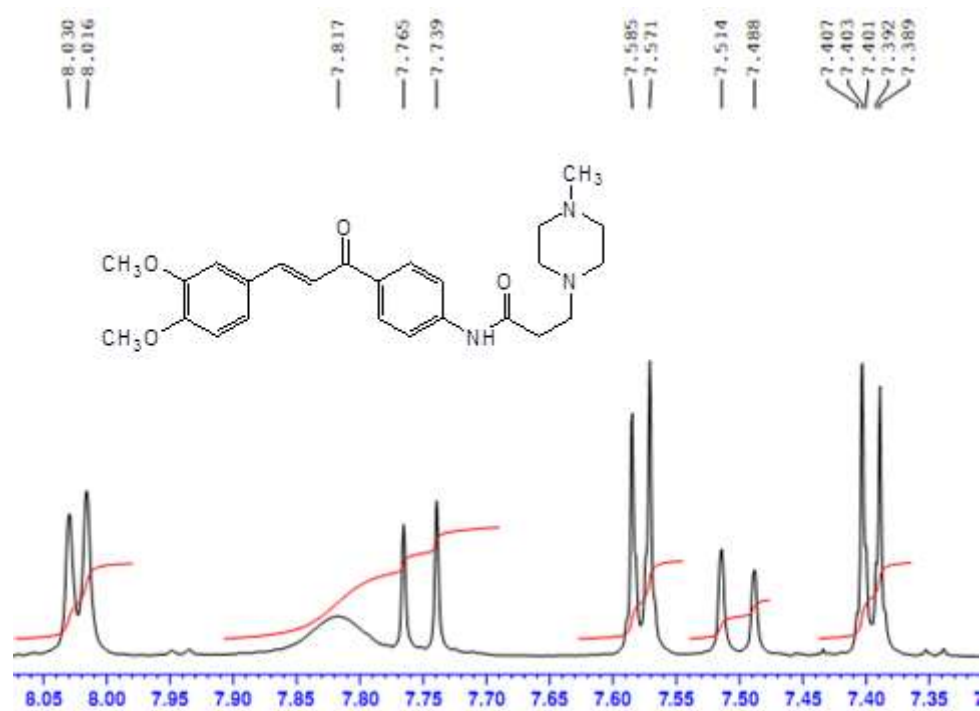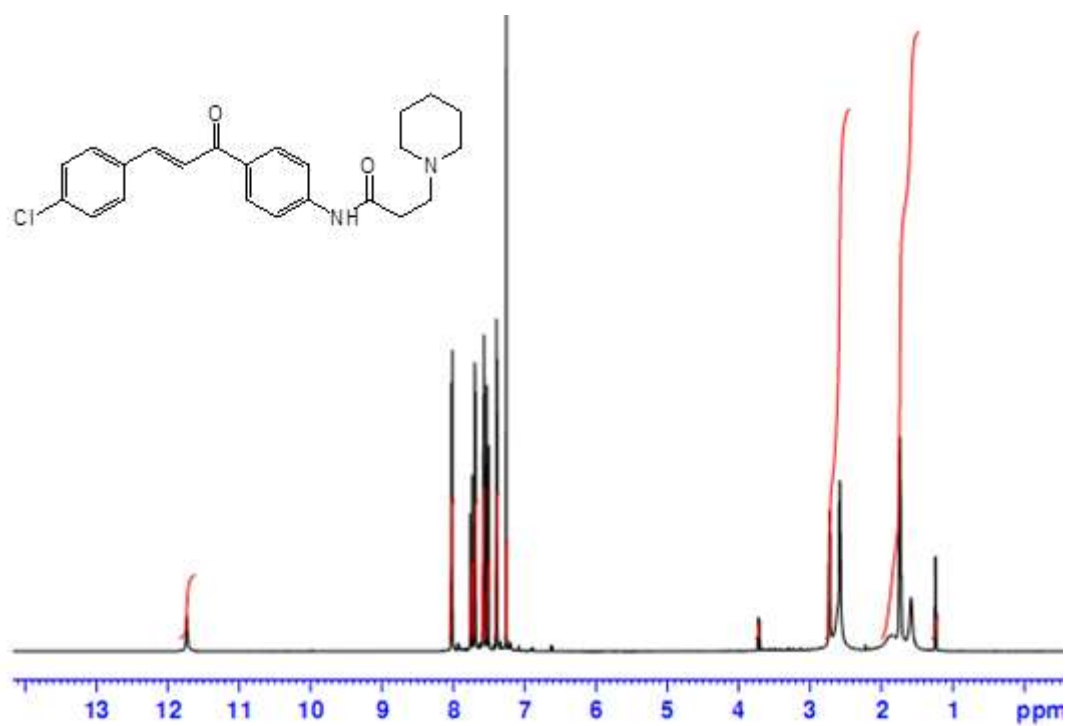

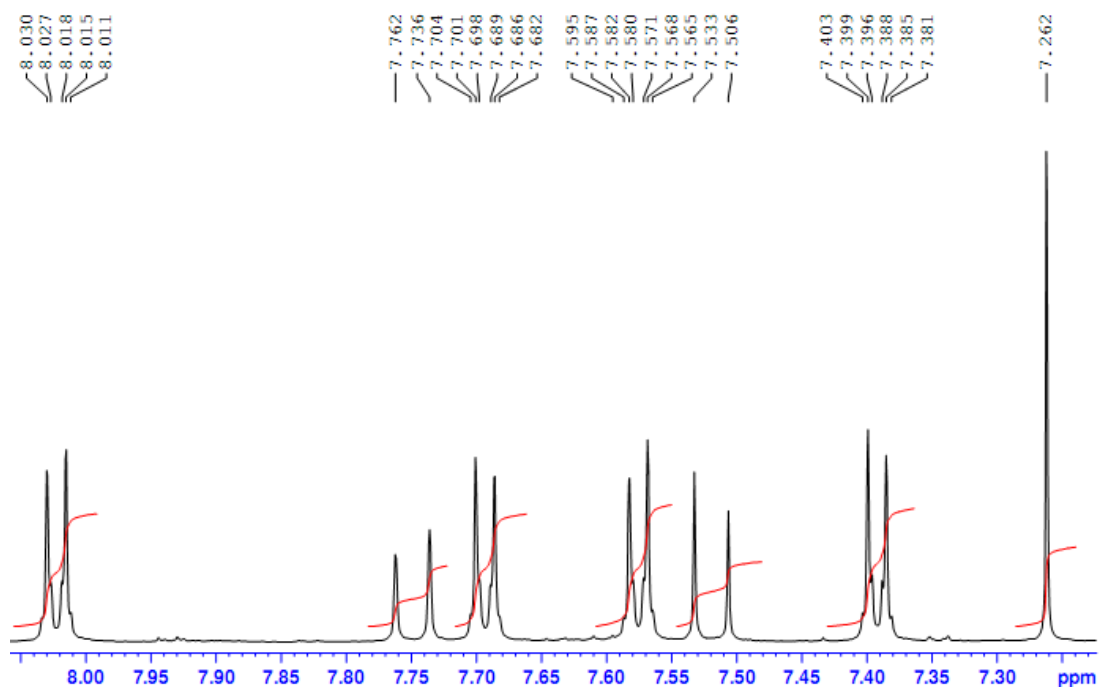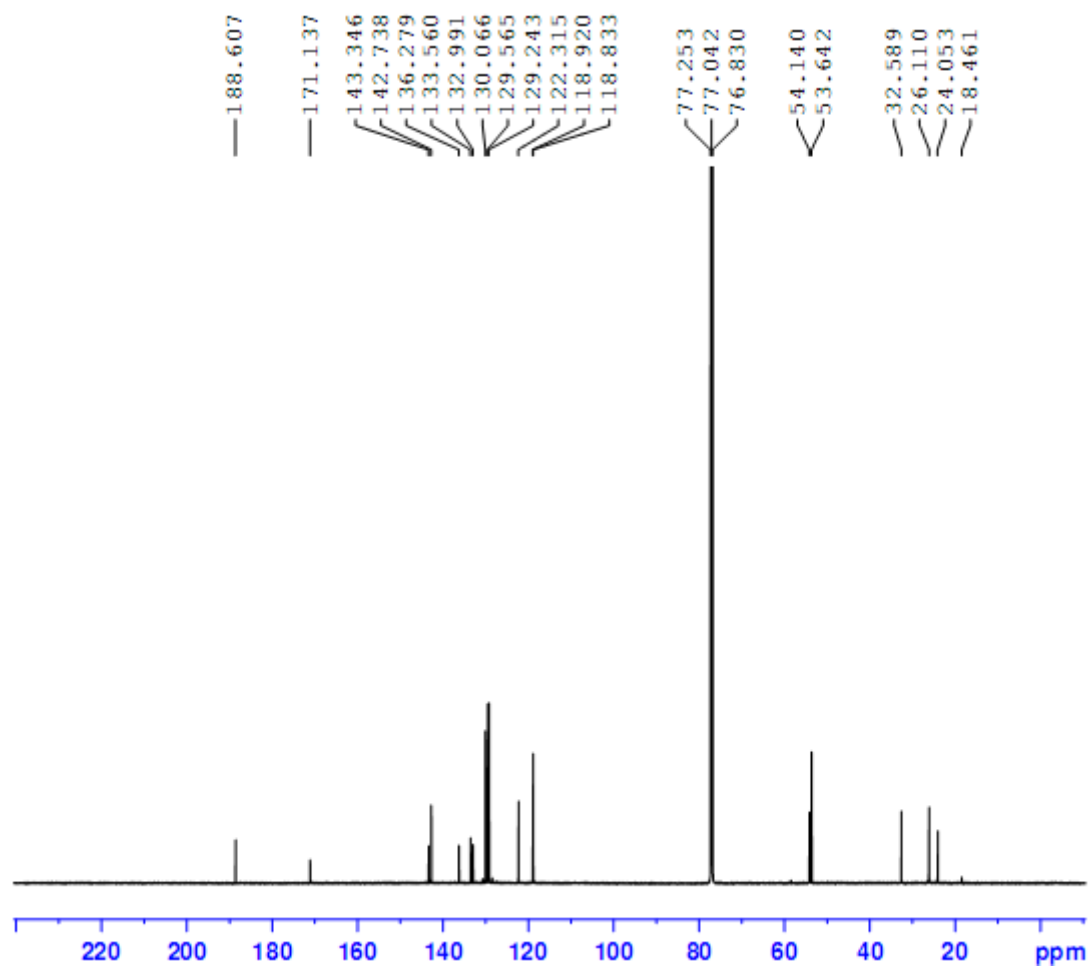

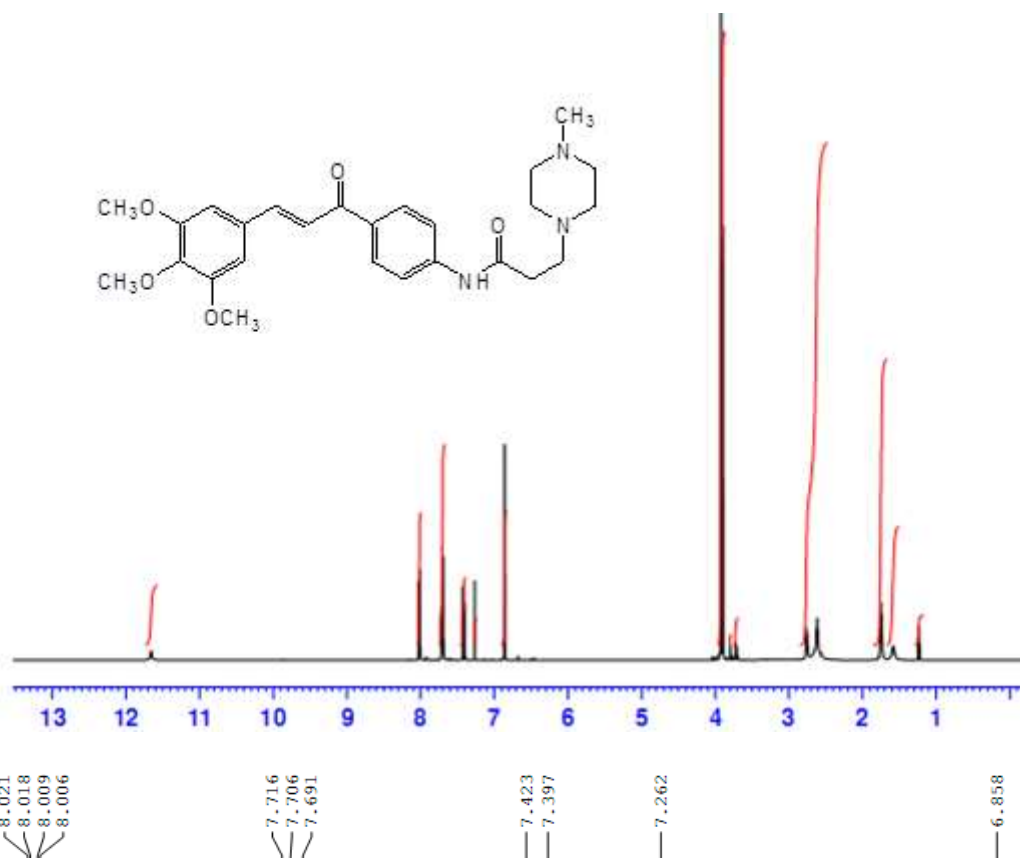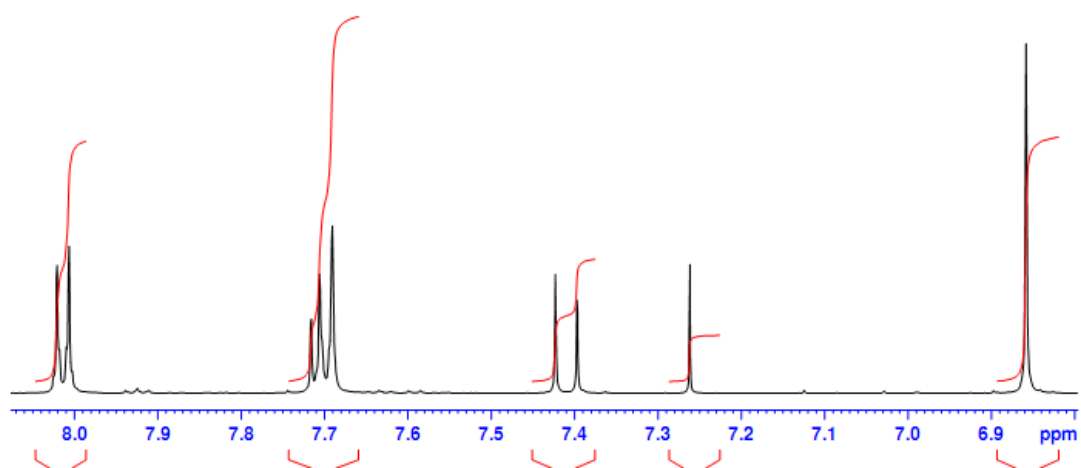

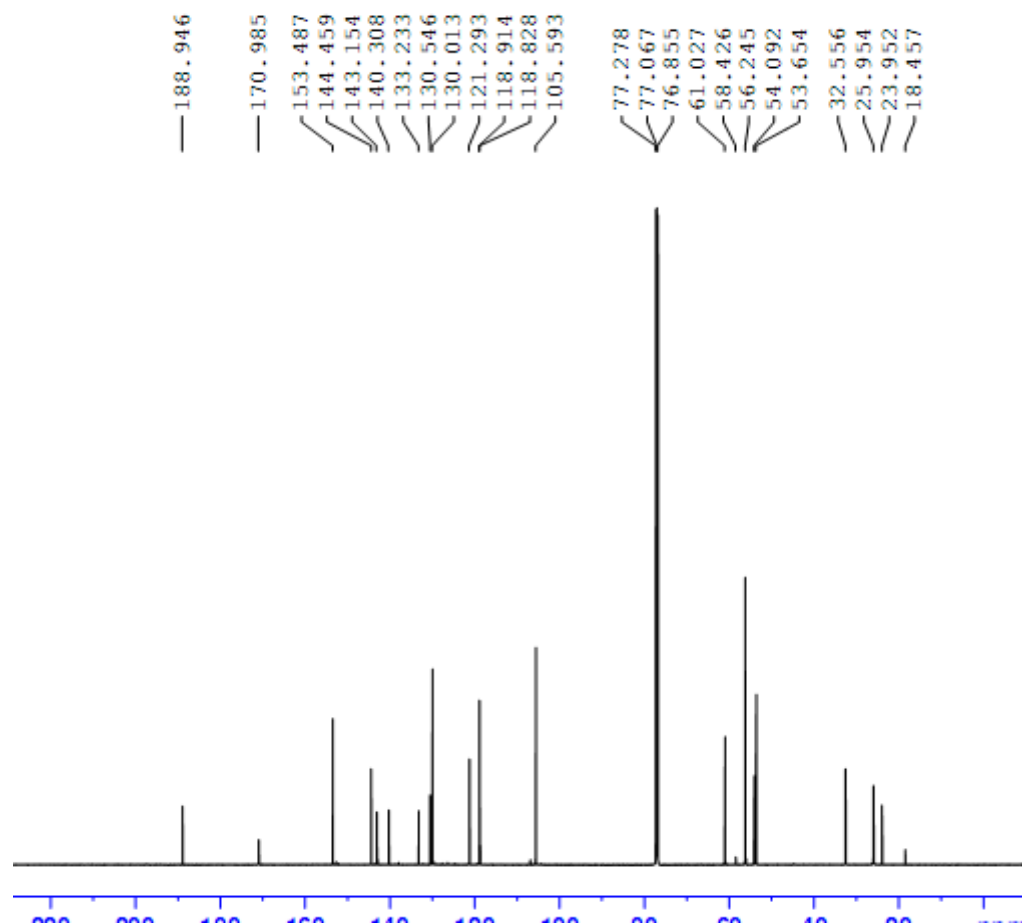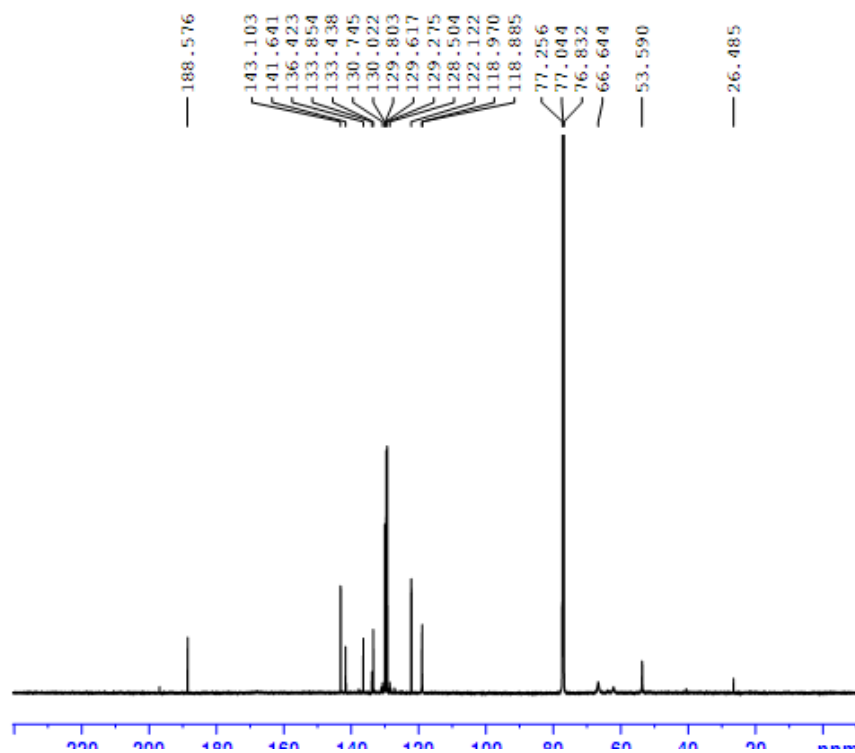

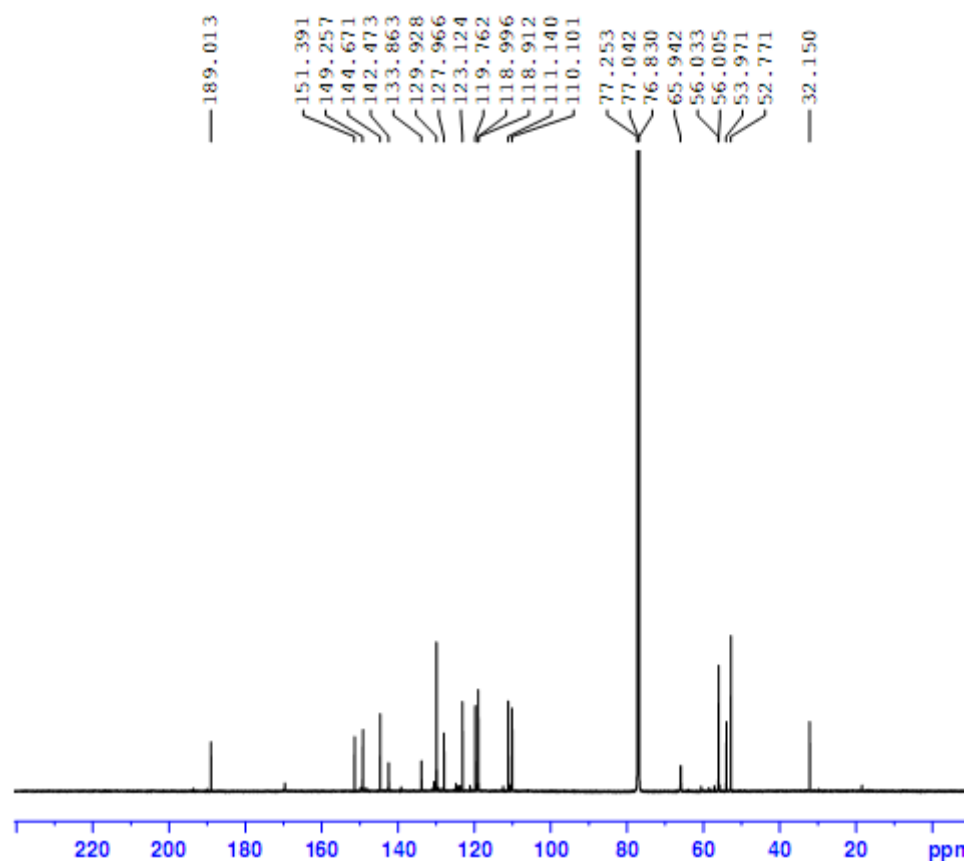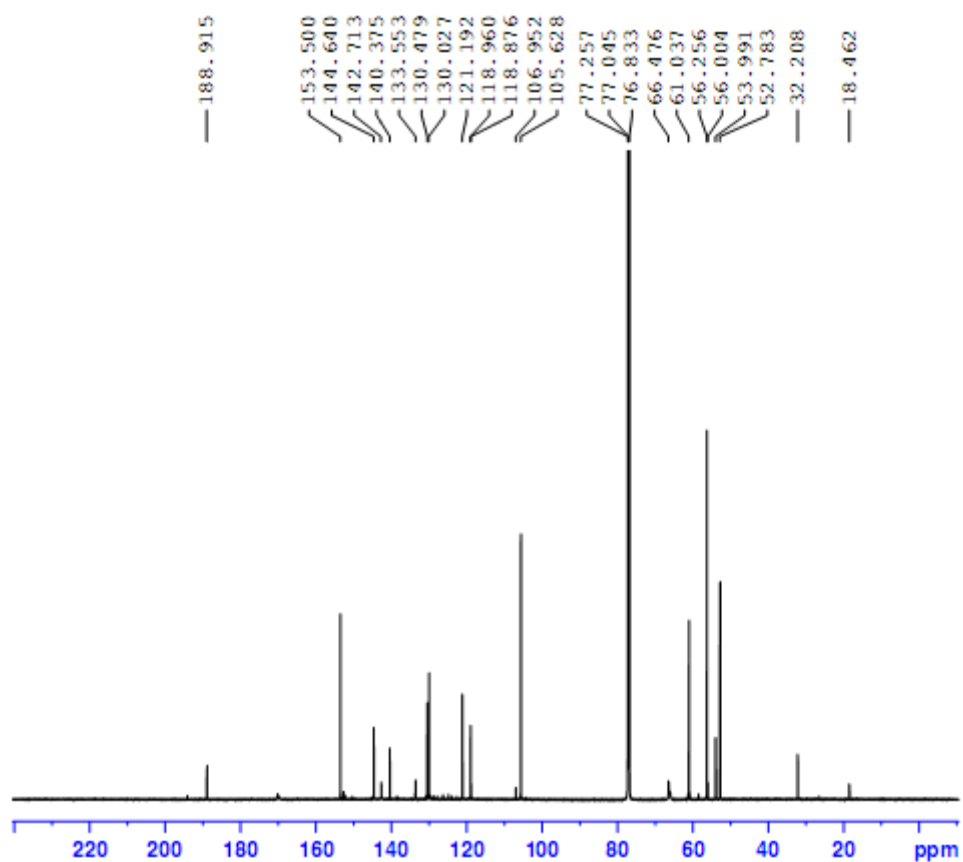

Supplement: IENZ_1461855_Supplementary_material.pdf [file IENZ_A_1461855_SM0963.pdf]
